# Study protocol for Sauti ya Vijana (The Voice of Youth): A hybrid-type 1 randomized trial to evaluate effectiveness and implementation of a mental health and life skills intervention to improve health outcomes for Tanzanian youth living with HIV

**DOI:** 10.1371/journal.pone.0305471

**Published:** 2024-08-26

**Authors:** Getrud J. Mollel, Eunice Ketang’enyi, Lilian Komba, Blandina T. Mmbaga, Aisa M. Shayo, Judith Boshe, Brandon Knettel, John A. Gallis, Elizabeth L. Turner, Karen O’Donnell, Joy Noel Baumgartner, Osondu Ogbuoji, Dorothy E. Dow

**Affiliations:** 1 Ifakara Health Institute, Ifakara, Tanzania; 2 Baylor College of Medicine Children Foundation of Tanzania, Mwanza, Tanzania; 3 Baylor College of Medicine Children Foundation of Tanzania, Mbeya, Tanzania; 4 Kilimanjaro Christian Medical Centre, Moshi, Tanzania, Moshi, Tanzania; 5 Kilimanjaro Christian Medical University College, Moshi, Tanzania; 6 Kilimanjaro Clinical Research Institute, Moshi, Tanzania; 7 School of Nursing, Duke University Medical Center, Durham, NC, United States of America; 8 Duke Global Health Institute, Duke University, Durham, North Carolina, United States of America; 9 Duke Department of Biostatistics & Bioinformatics, Duke University, Durham, NC, United States of America; 10 Center for Child and Family Policy, Sandford School of Public Policy, Durham, NC, United States of America; 11 School of Social Work, University of North Carolina at Chapel Hill, Chapel Hill, NC, United States of America; 12 Duke Center for Policy Impact in Global Health, Durham, NC, United States of America; 13 Department of Pediatrics, Infectious Diseases, Duke University Medical Center, Durham, NC, United States of America; Public Library of Science, UNITED KINGDOM

## Abstract

**Objective:**

Young people living with HIV (YPLWH) experience increased morbidity and mortality compared to all other age groups. Adolescence brings unique challenges related to sexual reproductive health, the elevated importance of peer groups, and often, emerging symptoms of emotional distress. Failure to address this unique life stage for YPLWH can lead to worse HIV and mental health outcomes. Herein lies the protocol for a hybrid-type-1 effectiveness-implementation trial designed to evaluate a mental health and life skills intervention that aims to address these needs for YPLWH in Tanzania.

**Methods:**

This is an individually randomized group-treatment trial designed to evaluate the effectiveness of Sauti ya Vijana (SYV: The Voice of Youth) toward improving viral suppression (HIV RNA <400 copies/mL) and mental health outcomes and to assess implementation including acceptability, feasibility, fidelity, and cost-effectiveness of the manualized intervention. The trial is being conducted across four geographically distinct regions of Tanzania. Peer group leaders (PGL) with lived HIV experience deliver the 10-session group-based intervention and two individual sessions during which participants describe their disclosure narrative (when they learned they live with HIV) and value-based goal setting. Caregiver or chosen supportive adults are encouraged to attend two specific group sessions with their youth. Participants are 10–24 years of age, prescribed antiretroviral therapy for at least 6 months, fully aware of their HIV status, able to commit to session attendance, and able to understand and meaningfully contribute to group sessions. Participant study visits occur at 5 time points for evaluation: baseline, 4-, 6-, 12-, and 18-months post baseline. A single booster session is conducted before the 12-month visit. Study visits evaluate mental health, adverse childhood events, interpersonal violence, resilience, stigma, HIV knowledge, substance use, sexual relationships, ART adherence, and HIV RNA. Implementation outcomes evaluate feasibility and acceptability through attendance, intervention session notes, focus discussion groups and qualitative interviews. Fidelity to the intervention is measured using fidelity checklists by a PGL observer at each group session. Cost effectiveness is calculated using an incremental cost-effectiveness ratio that utilizes a patient cost questionnaire and financial records of study costs.

**Significance:**

Few mental health interventions for YPLWH have demonstrated effectiveness. Results from this study will provide information about effectiveness and implementation of a peer-led intervention for delivering a mental health and life skills intervention in low-income settings.

**Trial identifier:**

This trial is registered at clinicaltrials.gov NCT05374109.

## Introduction

Reduction in AIDS related mortality has not been realized in young people living with HIV (YPLWH, 10–24 years of age) at the same rate as for other age groups. In Africa, 60% of the continent’s population is under the age of 25 years, and this is where a staggering 85% of the world’s YPLWH reside [1]. Globally, an estimated 1600 new HIV infections occur every day in youth 15–24 years of age; and a young person dies every 10 minutes due to AIDS-related illness [2], far more than any other age group. YPLWH are a unique and vulnerable population that harbor a high burden of global HIV infection and are a major contributor of HIV transmission. Innovative interventions tailored to their specific needs are urgently needed to combat these devastating outcomes and to prevent the spread of HIV.

The developmental period of adolescence brings tremendous hormonal, physical, and neurologic changes. These changes help explain the tendency for adolescents to engage in risk taking behavior, substance use, and the emergence of common mental disorders (ex. depression, anxiety, post-traumatic stress) [3]. In addition to dramatic biologic changes, the relevance of their social environments also change; that is, peer networks and “fitting in” become priorities and can compromise rational decision making [4, 5]. YPLWH have the added burden of navigating peer and romantic relationships while living with a stigmatizing, sexually transmissible infection that requires adherence to daily treatment. These are among many factors that contribute to delayed diagnosis, loss to follow-up, inadequate adherence to antiretroviral therapy (ART), and worse HIV outcomes compared to children or adults [6–10].

During adolescence, neurocognitive and pubertal maturation interact with social and environmental factors to create a highly dynamic health profile for young people [11]. Multiple social determinants of health are interconnected with complex relationships that may influence the development of emotional distress, psychopathology, and HIV health outcomes [5, 12–15]. The important influence of psychosocial factors (stigma, coping, supportive relationships, and other protective factors) on mental health and ART adherence is well documented [16, 17]; but there are few interventions tailored to YPLWH that address the pathway toward improved medication adherence and virologic suppression through improved mental health [17].

Failure to address the importance of mental health in this vulnerable and growing population of YPLWH threatens progress towards ending HIV/AIDS as a public health threat by 2030 [18]. It is well documented that virologic suppression (undetectable) prevents ongoing HIV transmission (untransmittable) [19], or “U = U” [20]. YPLWH represent a known and specific obstacle to realizing this goal given their risk for low ART adherence, active HIV replication, and emerging sexual activity. There remains a critical gap in evidence-based life skills and mental health interventions to meet the specific needs of YPLWH in Africa, where the largest burden of disease lies.

In response, Sauti ya Vijana (SYV: The Voice of Youth), is proposed as a highly promising mental health and life skills intervention that engages YPLWH and their caregivers in a peer-led, group-based intervention [21]. SYV was co-developed with and for YPLWH based on formative work [22–25] and has undertaken an extensive development and piloting phase (2016–2020) in Tanzania [26–29]. The original pilot trial demonstrated that the intervention was feasible, acceptable, and likely led to improved ART adherence, virologic suppression, and mental health for YPLWH [27]. In this manuscript, the protocol for the SYV hybrid type-I randomized controlled trial is presented and includes three aims: Aim 1: Evaluate SYV effectiveness to improve HIV RNA suppression among YPLWH in Tanzania; Aim 2: Elucidate the mechanism of change by exploring how and for whom is SYV intervention most effective; Aim 3: Evaluate implementation outcomes of acceptability, feasibility, fidelity, and cost-effectiveness across sites.

## Methods

### Trial setting

**Fig 1:**
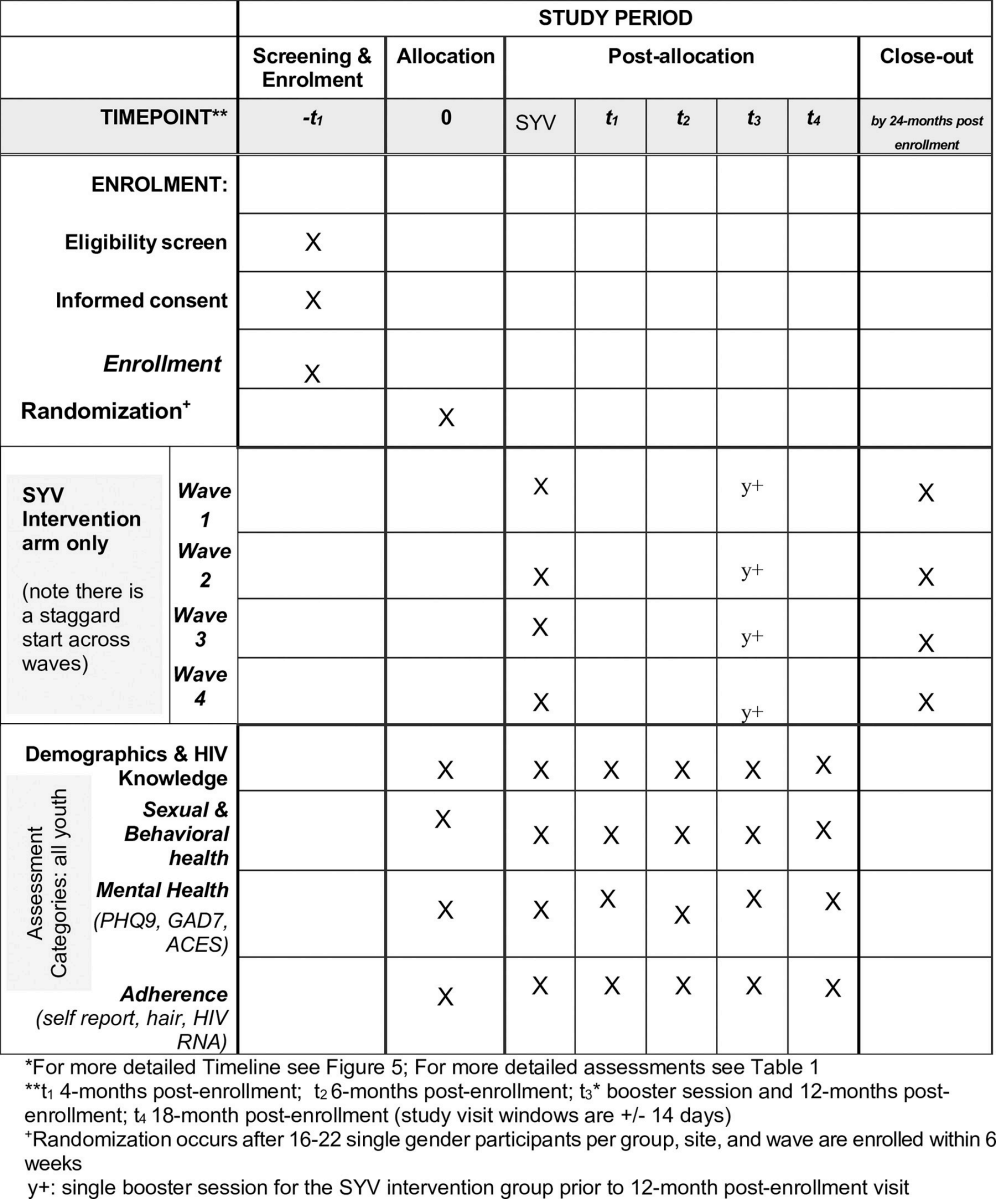
Spirit schedule.

**Fig 2:**
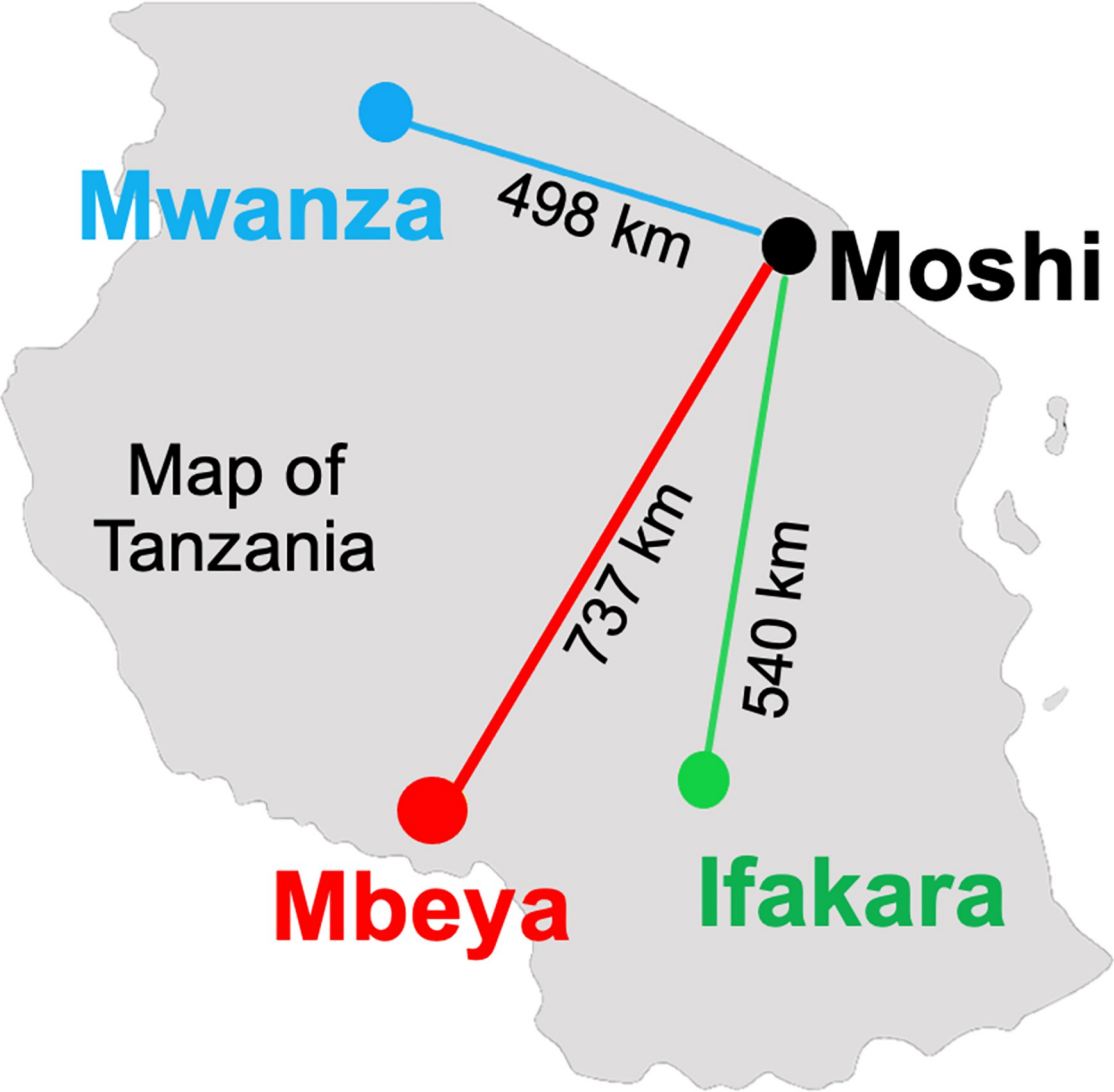
Tanzanian map reflecting distances for each study site location.

The trial takes place across four distinct regions of the East African country of Tanzania: Moshi-Kilimanjaro, the region where the original pilot was conducted; Ifakara-Morogoro region, Mbeya, and Mwanza (Figs 1 and 2). Sites were chosen based on four factors: (1) a high prevalence of adolescents living with HIV, (2) an adolescent HIV clinic with youth with unmet mental health needs, (3) research infrastructure that includes a quality assured laboratory to run HIV RNA, and (4) adequate classroom space to conduct the intervention sessions. All sites are referral facilities with a minimum population of 300 YPLWH that attend the adolescent HIV clinic.

### Design

The study design is a hybrid-type 1 effectiveness-implementation trial that first includes a preliminary, non-randomized, pre-trial phase of the intervention in all sites to orient the responsible teams on key study procedures and to practice the logistics of study visits and intervention delivery. Implementation adaptations that improve trial logistics are tracked using the Frame framework and implemented accordingly [30].

Then, a two-arm individually randomized group-treatment (IRGT) trial with clustering in the intervention arm due to the group-based nature of the SYV intervention is conducted. Adolescents receive information about the SYV intervention. Those who meet eligibility criteria and provide consent enroll and are randomized to receive standard of care or the SYV intervention. Those in the SYV intervention arm have a home visit whereby the peer group leader (PGL) re-introduces the participant and their caregiver(s) to the goals and content of SYV. The youth meet in groups of 10–11 participants for 10 consecutive weekly meetings and 2 individual sessions. Caregivers (or another supportive adult) are asked to participate in the first and the sixth sessions. The intervention arm ends with a final review at the end of the 10 group sessions and a repeat “booster” review session before the 12-month study visit. Participants randomized to the standard of care (SOC) arm continue to receive usual clinical care that includes routine adolescent peer programs and treatment visits. The mechanism of change is detailed below with implementation outcomes carefully evaluated using the CFIR framework [31].

### Study population

The study enrolls YPLWH between the ages of 10 and 24 years who attend one of the four adolescent HIV clinics included in the study, are fully disclosed and aware of their HIV status, have been prescribed ART for a minimum of 6 months, and can commit to attending the 10 group and 2 individual sessions. YPLWH are grouped by gender and age for the 10 SYV sessions.

Exclusion criteria include any one of the following: active psychosis or cognitive disability that precludes active participation in consent process, intervention, or assessment interviews as determined by the study principal investigator or research assistant involved in the consent process.

**Fig 3:**
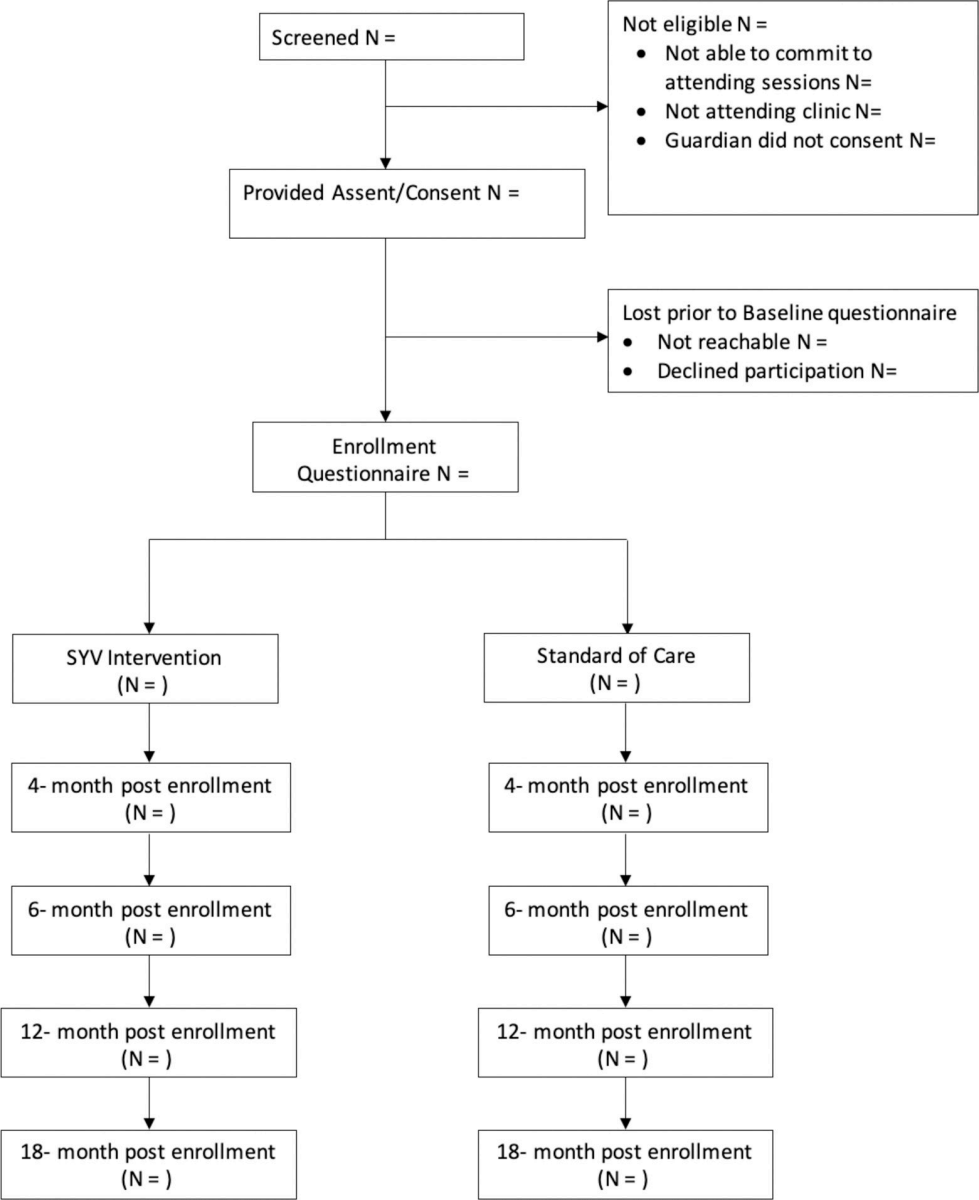
Consort diagram flow.

The study is designed to recruit and enroll participants from clinics serving YPLWH within each of the four Tanzanian study sites. Approximately 64 youth are enrolled for the preliminary, non-randomized, pre-trial phase (eight males and eight females per site) and approximately 175 participants from each of the four Tanzanian study sites (N = 700). The flow of participants through the RCT is shown in Fig 3.

### Consent

Participation in the study is voluntary, and each participant can choose to leave the study at any time for any reason without penalty. A trained research assistant obtains written, informed consent from all participants. For those under 18 years, written participant assent is obtained in addition to written caregiver/guardian consent. The caregiver/guardian must be at least 18 years old and accompany their youth for the consent/assent process. Participant and caregiver travel is reimbursed for the consent process during which the purposes and risks of the study are explained. If the caregiver/guardian is unable to come to the clinic in person, informed consent can be discussed through a home visit or by a telephone call between the research assistant and caregiver. In the latter case, the caregiver/guardian then signs the consent form and returns the form back with the participant.

### Intervention

The intervention, Sauti ya Vijana (SYV, The Voice of Youth), was developed and specifically tailored to the needs of YPLWH in Tanzania [21]. Building on the well-established findings that mental health is associated with ART adherence and HIV outcomes [32], SYV was designed to address the challenges identified in the formative research [22, 24]. As shown in the preliminary work, 32% of YPLWH reported symptoms of mental health difficulties (symptoms of depression, post-traumatic stress, and/or other emotional/behavioral problems) that were associated with how participants learned that they live with HIV (primary HIV disclosure), stigma, and worse self-reported treatment adherence [22, 25]. To reach a large number of youth while building on the communal aspect of Tanzanian culture and the importance of peers, SYV is a group intervention adapted, in part, from a previous trauma-focused cognitive behavioral therapy study with HIV-affected Tanzanian children who experienced parental death [33, 34].

**Fig 4:**
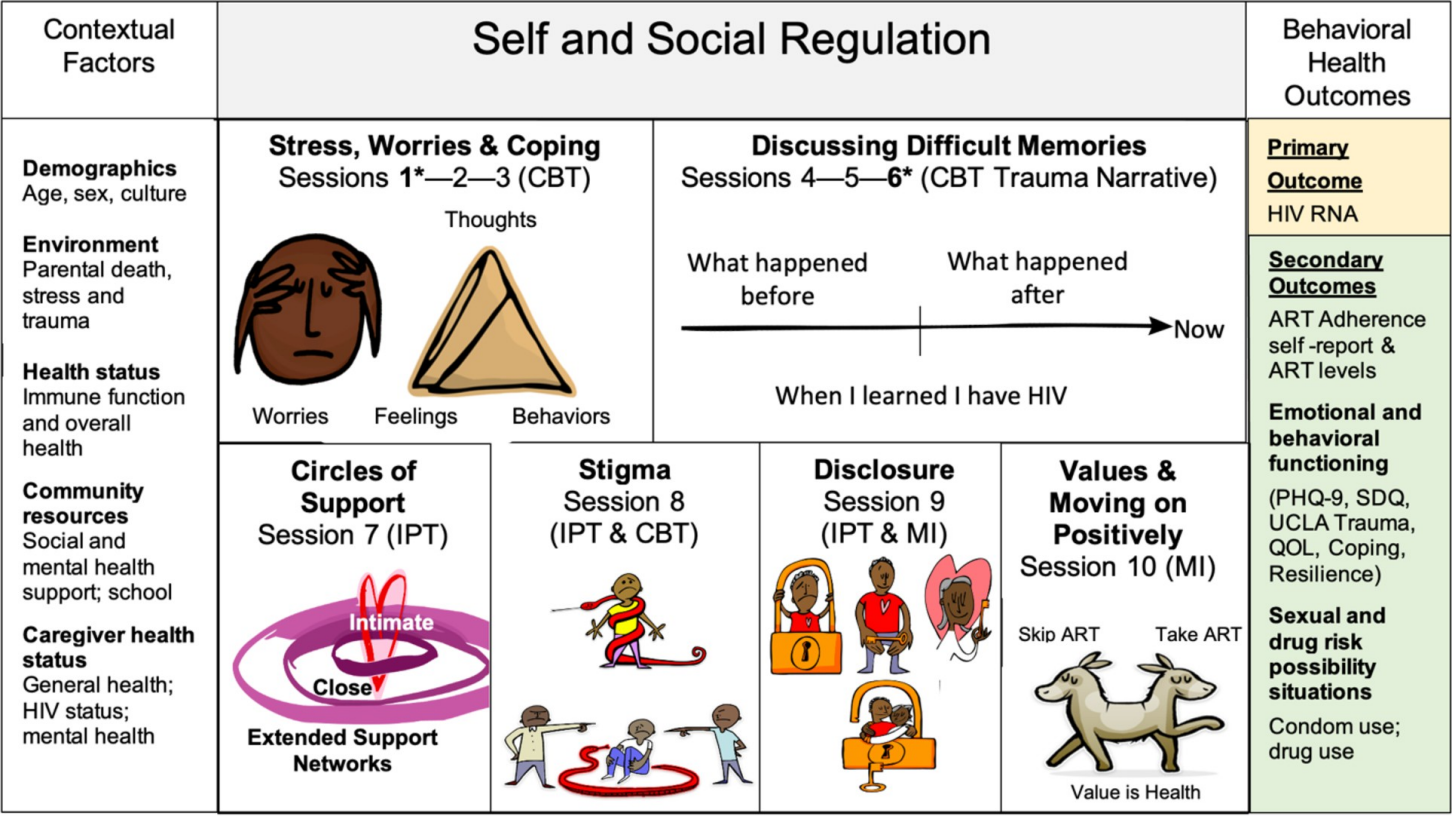
Sauti ya Vijana (SYV) applied to the modified social action theory framework^35^. Abbreviations: Psychotherapeutic strategies informed by 1) CBT: Cognitive Behavioral Therapy; 2) IPT: Interpersonal Psychotherapy: 3) MI: Motivational Interviewing; ART: antiretroviral therapy; PHQ: Patient Health Questionnaire; SDQ: Strengths and Difficulties Questionnaire; QOL: quality of life *Two sessions (one and six) are joint with caregivers; Not shown are two individual sessions (after session 4 and after session 10). Republished from Sauti ya Vijana (https://sites.duke.edu/sautiyavijana/about-syv/) under a CC BY license, with permission from Dorothy Dow, original copyright 2020.

SYV includes ten group sessions (two held jointly with caregivers) each lasting approximately 90–120 minutes and two individual sessions delivered by trained PGL (23–29 years of age and living with HIV) using a manualized protocol. The intervention builds upon the modified Social Action Theory Resilience Framework for YPLWH [35] and strategically uses components of evidence-based treatment models to influence cognitive, self, and social regulation to improve behavioral health outcomes (Fig 4). The SYV intervention integrates components of three psychotherapeutic models: Trauma-Informed Cognitive Behavioral Therapy (TI-CBT) [36], which in the developing brain is thought to promote cognitive restructuring and influence connectivity from the amygdala towards the prefrontal cortex, an area associated with planned behavior and reasoned decision-making [37]; Interpersonal Psychotherapy (IPT) [38], which is designed to improve social relationships, especially as support for traumatic experiences and life transitions; and Motivational Interviewing (MI) [39] with individual value-guided goal pursuit [40]. During this pivotal developmental period of adolescence when critical foundations of self and social regulation are realized, SYV is created to prevent or reduce the severity of common mental disorders for this group of youth, thereby improving health outcomes [41].

### Training and supervision

**Fig 5:**
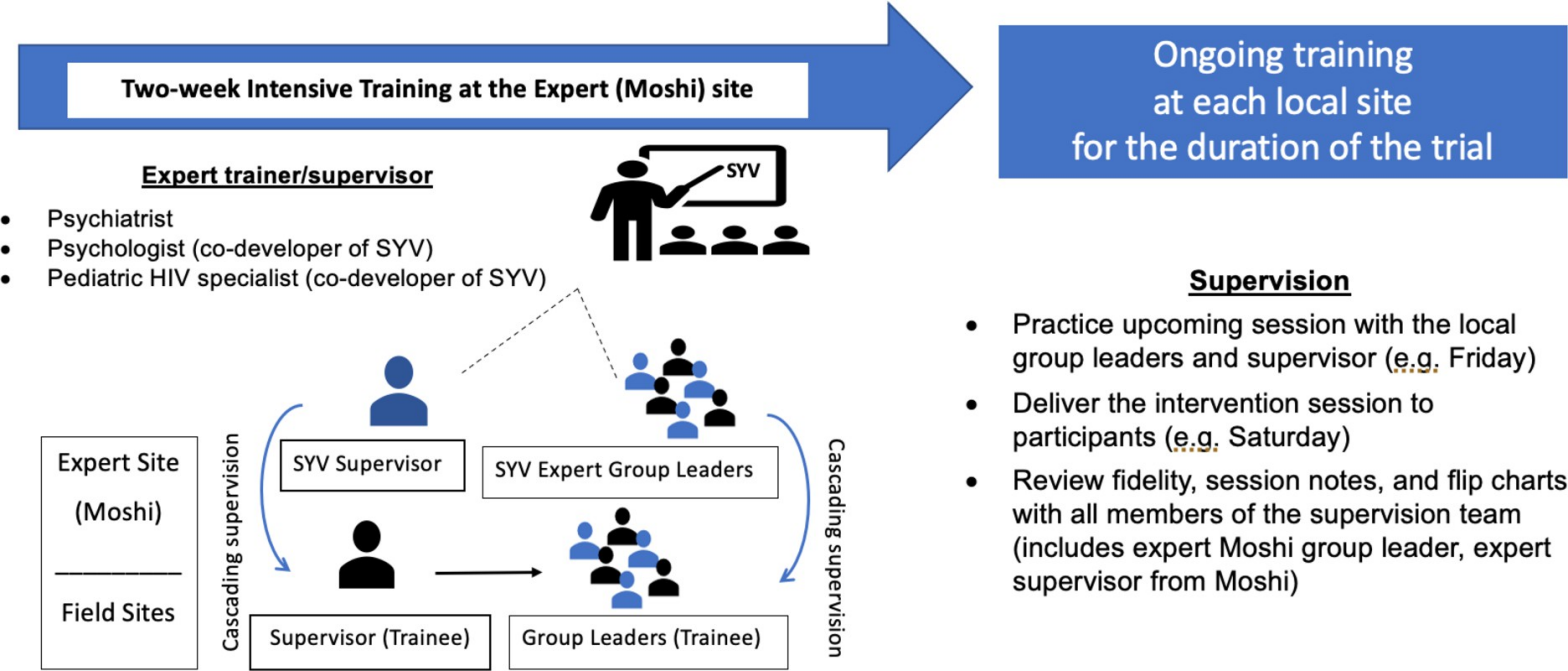
SYV training and supervision.

The intervention is designed from a task sharing model and is delivered by trained young adults in group-based, gender concordant sessions. Each site team is comprised of six to seven peer group leaders, half male and half female. Group leaders were nominated by the clinical staff from their adolescent HIV clinic and interviewed for the role with proficiency in reading, writing, and exhibiting confidence and good medication adherence. Group leaders from all the sites met in Moshi, Kilimanjaro for an intensive 11-day training prior to any study activities. During training, they learned the basics of research ethics, group dynamics, group facilitation skills, and the theory behind the three psychotherapies incorporated into SYV. Expert group leaders who delivered the original SYV pilot study in Kilimanjaro demonstrated session delivery. Then, group leaders in each of the four sites practiced each session within their team with their supervisor in breakout sessions. Two group leaders practiced facilitating the sessions and one group leader documented fidelity and participant engagement. Group leaders continue to practice the manual before session delivery throughout the trial (Fig 5).

The supervision team consists of an expert group leader (a young adult who helped develop, adapt, and deliver SYV in the original Kilimanjaro pilot and has since aged out of the role), a local site supervisor (a clinical counselor or nurse psychiatrist), and an expert mental health specialist with a doctoral degree in psychology or psychiatry (from USA or Tanzania). The supervision team ensures intervention fidelity by providing the group leaders feedback during the practice sessions, encouraging consistent content delivery, and emphasizing adherence to the manualized protocol. In the actual session, group leaders self-report intervention fidelity using a fidelity checklist. They document the time required to teach each component; and they document questions, responses, and examples provided by from participants that demonstrate understanding of the material. Reports are reviewed during weekly supervision and include 1) the submitted checklists used to document fidelity to the protocol, 2) session notes that document group leader observations and interactions with each participant, and 3) the flip charts used to teach each session. The supervision team is always available to respond to any safety concerns that arise in group and also support group leaders who are experiencing any of their own vicarious trauma or other mental health symptoms. A bi-weekly supervision meeting is held with the local supervisor who observes the group leaders practice the upcoming session (Fig 5).

### Standard of care

The standard of care (SOC) at all four sites is similar with all offering a local adolescent-specific HIV clinic within the larger HIV Care and Treatment Center. All sites offer free ART, with most youth receiving tenofovir, lamivudine, dolutegravir (TLD) regimen, and HIV RNA SOC testing at the government laboratory. With multi-month dispensing provided at all sites, youth with HIV RNA <50 copies/mL receive 3 to 6 months of medication (pending supply) and clinic appointments every six months with annual HIV RNA testing [42]. Youth with ≥50 copies/mL are scheduled for monthly medication refills, education, and adherence counseling. After three rounds of adherence counseling the HIV RNA is re-checked. The result determines whether the youth will transition to multi-month dispensing (HIV <50 copies/mL) or continue with monthly appointments (if HIV RNA remains > 50 copies/mL).

Youth enrolled in SYV and randomized to the intervention plus SOC arm share a clinic home with youth randomized to the SOC only arm. Due to potential participant mixing, the study visit questionnaires ask about exposure to intervention content in an attempt to assess possible contamination between study arms. Participants randomized to the SOC arm continue to receive routine care as usual. The SOC arm does not meet in groups as part of the study, but continue to meet in routine clinic-sponsored adolescent HIV peer groups available across all clinic sites. Attendance data from these clinic-sponsored groups are available, but are not being tracked per study protocol given the possibility of youth from both study arms interacting not only in the clinic peer groups, but also in school, in their neighborhood, and beyond the clinic infrastructure.

### Outcome measures

Data are collected at five time points corresponding to baseline (T_0_) and the following four time points after baseline: 4 months (T_1_), 6 months (T_2_), 12 months (T_3_), and 18 months (T_4_), with a window around each time point of +/- 14 days. Primary and secondary outcome measures are summarized in Table 1.

**Table 1:** SYV study outcomes.

Domain	Construct	Tool	Specifications	Administration				
				**T**_**0**_	**T**_**1**_	**T**_**2**_	**T**_**3**_	**T**_**4**_
Primary Outcome	Virologic Suppression	Viral load (HIV RNA <400 copies/mL)	Binary	□	□	□	□	□
Secondary Outcomes	Log viral load	HIV RNA copies/mL)	Continuous	□	□	□	□	□
	Adherence	Self-report, 3 questions [43]	Continuous	□	□	□	□	□
		ART levels in hair [44]	Continuous	□		□		
	Behavior	Sexual activity and condom use	Binary	□	□	□	□	□
		Substance use	Binary	□	□	□	□	□
	Mental Health	Patient Health Questionnaire (PHQ-9) [45, 46]	Continuous and binary	□	□	□	□	□
		Adverse Childhood Events-International Questionnaire and UCLA Trauma Reaction Index survey [47, 48]	Continuous and binary	□	□	□	□	□
		Generalized Anxiety Disorder-7 Questions (GAD-7) [49]	Continuous and binary	□	□	□	□	□
	Coping	The brief COPE [50]	Continuous	□	□	□	□	□
	Resilience	People Living with HIV Resilience Scale [51]	Continuous	□	□	□	□	□
	Stigma	10 question stigma scale [52]	Continuous	□	□	□	□	□
	Quality of Life	WHOQOL-HIV-BREF [53]	Continuous	□	□	□	□	□
	Gender based violence	WHO Victim/Perpetrator [54]	Continuous	□	□	□	□	□
	Disclosure	Purposefully disclosed to someone new (if yes, who and why)	Binary	□	□	□	□	□
	HIV knowledge	HIV knowledge [55]	Continuous	□	□	□	□	□

*T_0_: Baseline; T_1_: 4 months; T_2_: 6 months; T_3_: 12 months; T_4_: 18 months

### Objectives and hypothesis

The *central effectiveness hypothesis* is that SYV will increase virologic suppression in YPLWH in Tanzania. SYV is hypothesized to improve the secondary outcomes listed in Table 1, including ART adherence and mental health outcomes; and the intervention will be acceptable, feasible, delivered with fidelity, and cost effective.

### Randomization

Approximately 700 participants will be randomized across the four sites to receive the SYV intervention arm or SOC arm. Enrollment began on 1 March 2023 and is expected to be completed December 2024. Randomization occurs by computer-generated permuted blocks of size 4 generated using SAS software, is performed separately at each site, and is stratified by sex and enrollment HIV RNA (<400 copies/mL or ≥400 copies/mL) to ensure balance on these variables. The randomization is implicitly stratified by site, and that all groups are single gender. Those randomized to receive the SYV intervention will be assembled into 33 groups of 10–11 participants per group, across 4 sites. The outcomes assessor and statisticians will be masked to participant study arm until the completion of all 6-month study visits (primary outcome).

### Sample size

**Fig 6:**
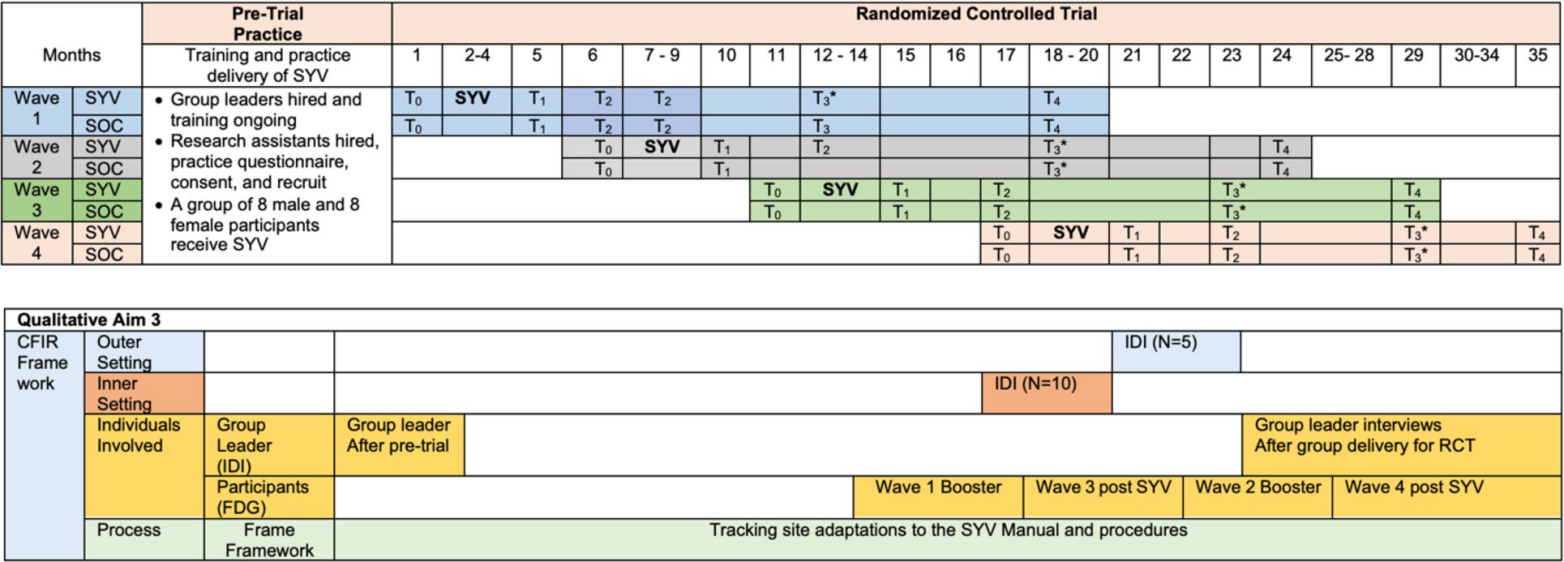
Study timeline. Effectiveness Aim 1: SYV: Sauti ya Vijana (Voice of Youth) Intervention delivery occurs over 12 weeks. T_0_ is enrollment study visit; T_1_ is 4-month; T_2_ is 6-month and the study primary endpoint; T_3_*includes a one-session booster before the 12-month visit; T_4_ is 18-month study visit. Qualitative Aim 3: IDI: in-depth-interview; FDG: Focus Discussion Group; Inner Setting, Outer Setting, Individuals involved.

Sample size is based on a plausible percentage of 80% viral suppression (VS) in the SOC arm and 90% in the SYV intervention arm at 6-months post-baseline (T_2_). This is a higher percentage than in our original pilot trial [56] because more effective ART medication (dolutegravir) and other factors are expected to lead to increased VS rates. An intra-cluster correlation (ICC) of 0.04 (similar to the original pilot data) is assumed; and because of possible dropout, 8 participants per group in the SYV intervention are expected to have data available for analysis at T_2_. With these assumptions, we have 90% power at a two-tailed significance (alpha) level of 0.05 to detect this 10-percentage point effect with 264 participants (33 groups of 8 participants each) in intervention and 311 participants in SOC (575 participants). Assuming 10% attrition by the primary time point in both arms (noting the 91% retention rate in the pilot trial) [27], the group size in intervention is increased to 9 (297 participants in 33 groups) and the number of participants in SOC to 346 for a final total sample size of 643 across both arms (Table 2). Although the power formula [57] used indicates unequal sample sizes (i.e., larger sample size in SOC), the number of participants in each group in the intervention arm is increased to 10–11 (49 additional participants spread across the 33 groups), eliminating the imbalance between arms, so that there are 346 participants in intervention and 346 in control. Data collection is expected to take place over 24-months to reach the primary endpoint and over 36 months for completion of all timepoints (Fig 6). Should attrition exceed 10% in the early waves, the sample size will be increased to ensure that evaluation of intervention effectiveness is appropriately powered.

**Table 2:** Power for expected effect size for primary outcome at 6-month time point.

	Initial, unequal N per arm	Final, equal N per arm
Power	90%	>90%
Alpha	5%	5%
Dropout Rate	10%	10%
Proportion VS in SYV Intervention	90%	90%
Proportion VS in	80%	80%
Intra-cluster correlation (ICC)	0.04	0.04
Number of participants per group	9	10–11
Number of participants in SYV	297	346
Number of participants in SOC	346	346
Total Number of participants*	**643**	**692**

*Number recruited and randomized; Abbreviations: VS–Virologically Suppressed; SOC–Standard of care (control)

#### Data collection, management, and analysis

*Data collection methods (quantitative)*. During recruitment, the local site teams identify and approach eligible YPLWH from study presentations and announcements during routine adolescent HIV clinics and through use of local clinic electronic medical records. Research assistants track recruitment status for potential participants in paper log books stored securely at the study sites. Screening, baseline, and follow-up survey data are collected using REDCap, a secure, web-based software platform designed to support electronic data capture for research studies [58, 59]. Specifically, these data are collected using the REDCap Mobile Application running on Android tablets, which allow data to be collected even when not connected to the internet.

Data collectors are trained on REDCap Mobile and practice data collection before and during the preliminary, non-randomized, pre-trial phase to ensure proficiency using data systems before any data are entered for the trial. Survey instruments include demographics, validated mental health questionnaires, ART adherence, substance use and sexual behavior questionnaires (Table 1) translated into the local language, Kiswahili, and back-translated to English to ensure conserved meaning. The REDCap software is programmed to notify study personnel by email of any participant reporting suicidal thoughts, moderate to severe symptoms of depression (PHQ-9 ≥10), symptoms of anxiety (GAD-7 ≥10), or reports of sexual abuse. The research assistants notify the study supervisor of any concern and ensure same day referral for urgent matters (suicidality; safety concerns) and referral within the week for elevated symptoms of mental distress. Participants with any mental health concern are tracked using a referral log book to ensure a referral was made and to document the outcome. This information is then keyed into the REDCap database for safety monitoring and evaluation and is reviewed quarterly by the data safety and monitoring board committee.

At baseline and all subsequent data collection time points, physical hair and blood samples are collected. Blood samples are sent to the quality assured laboratory at the study site for HIV RNA analysis. Participants have the choice to opt in to blood sample storage for future research, such as for resistance mutation testing. Hair samples are sent to the University of California San Francisco in the United States to evaluate for ART concentration in the body. Lab results are entered into REDCap. HIV RNA study results are provided to the participant’s clinician to be incorporated into their medical record.

*Data collection methods (qualitative)*. Qualitative data collection utilizes in-depth Interviews (IDIs) to capture individual experiences and focus discussion groups (FDGs) for information about the trial implementation science objectives related to acceptability, feasibility, and considerations of sustainability among key stakeholders. Key stakeholder IDIs and FGDs occur across the listed domains using the CFIR implementation science framework [31] (see Fig 6). IDIs and FDGs are conducted by trained Tanzanian qualitative researchers in the participant’s preferred language, Kiswahili or English. The IDIs and FDGs use semi-structured interview guides derived from domain maps tailored to the objectives of each stakeholder group and audio-recorded with participant consent. Data collected in Kiswahili are translated to English. The intervention session fidelity form, group leader session notes, flip chart images, and supervision notes are used to provide multiple measures about how each SYV session was received by the youth, the questions that were asked, responses provided, and to track participant attendance. Any notable site adaptations of the intervention are identified using the FRAME framework [30].

### Data management

#### Quality

To promote data quality, range and format, restrictions are hardcoded into the REDCap data collection instruments as appropriate for individual questions. Quality control reports are generated daily for REDCap data and returned to the data collection team for follow-up. Actions taken in response to quality control reports are transparently documented in code with supporting documentation, rendering all data processing reproducible.

#### Security and storage

Quantitative study data are collected and managed using REDCap electronic data capture tools hosted at Duke University. Data collected in REDCap are exported to and stored in Duke’s instance of Box, the cloud-based file storage software (DukeBox). All qualitative data from the IDI and FDG, fidelity forms, session notes, flip chart images, and supervision notes are uploaded and stored in DukeBox. The Duke installation of REDCap, the REDCap Mobile Application, and DukeBox are all approved by Duke Information Technology Security Office (ITSO) for secure storage of sensitive health data.

*Data access and governance*. A data transfer agreement and a material transfer agreement for hair samples are signed across all sites with investigators in accordance with the Tanzania national policies governing cross-border transmissions of research data and specimen. All investigators and the program manager have access to all study data.

### Statistical methods

#### Data analysis plan

*AIM 1 (Effectiveness of SYV)*. Results of statistical analysis will be reported according to the CONSORT guidelines [13, 14]. A flow chart will show the participation of both intervention and control arms in terms of screening, consent, baseline study visit, randomization and follow-up status (Fig 3). All analyses will focus on comparison of the two arms. Characteristics of participants will be reported by study arm.

*Analysis of primary and secondary outcomes*. The primary and secondary analyses are designed as intention-to-treat. Modified Poisson generalized estimating equation (GEE) models will be used to examine the effect of intervention on virologic suppression at each follow-up time point. To this end, the model will include intervention arm, follow-up time point modeled as a factor, and the interaction between intervention arm and time point. The GEE models will take into account clustering using an exchangeable working correlation matrix in the SYV intervention arm, and use small-sample corrected standard errors to take into account potential small-sample bias in the standard errors [60]. GEE-Matrix-adjusted Estimating Equations (GEE-MAEE) may be used to more suitably model the correlation structure across time within individuals and within SYV group (in the intervention arm) [61], although some research indicates that taking into account one level of clustering is sufficient [62].

For continuous secondary outcomes, linear mixed effects models will be used to include a random intercept for group, where each individual in the SOC arm is considered a group of size 1 [63], although GEE methods may also be used for continuous outcomes. Constrained longitudinal models that assume the intervention arm means are equal at baseline will be used [64]. For mixed models, type I error will be taken into account. It can be inflated when standard denominator degrees of freedom are used for hypothesis testing and when the number of groups is less than 40. In this case, the degrees of freedom will be adjusted for the hypothesis tests [65]. All models will be adjusted for the randomization stratification variables [66, 67]. Missing data will be considered by first examining baseline variables determined to be differential by dropout. These variables will then be included in the statistical models as a sensitivity analysis, under the assumption that the data are missing at random, conditional on the baseline variables. Sensitivity analyses will be conducted on differing cutoff values for HIV RNA suppression, namely 200 copies/mL and 50 copies/mL in addition to the primary endpoint cut off of 400 copies/mL at 6-months. Additionally, compliance (i.e., number of sessions attended in intervention arm) and spillover (i.e., amount of information from group sessions that control participants learn based on contamination questions included in the study visit questionnaire) will be measured. Causal inference methods will be used to examine the complier average causal effect (CACE), as well as the effect of spillover.

Effect modification will be analyzed by including each moderator separately (age, sex, site, and severity of mental health symptoms at baseline) in the statistical model with virologic suppression as the outcome, including the interaction between intervention arm and the moderator. Exploratory analyses of participants randomized to SYV will be conducted to compare outcomes according to degree of attendance to generate hypotheses around potential dose-response relationships.

**Fig 7:**
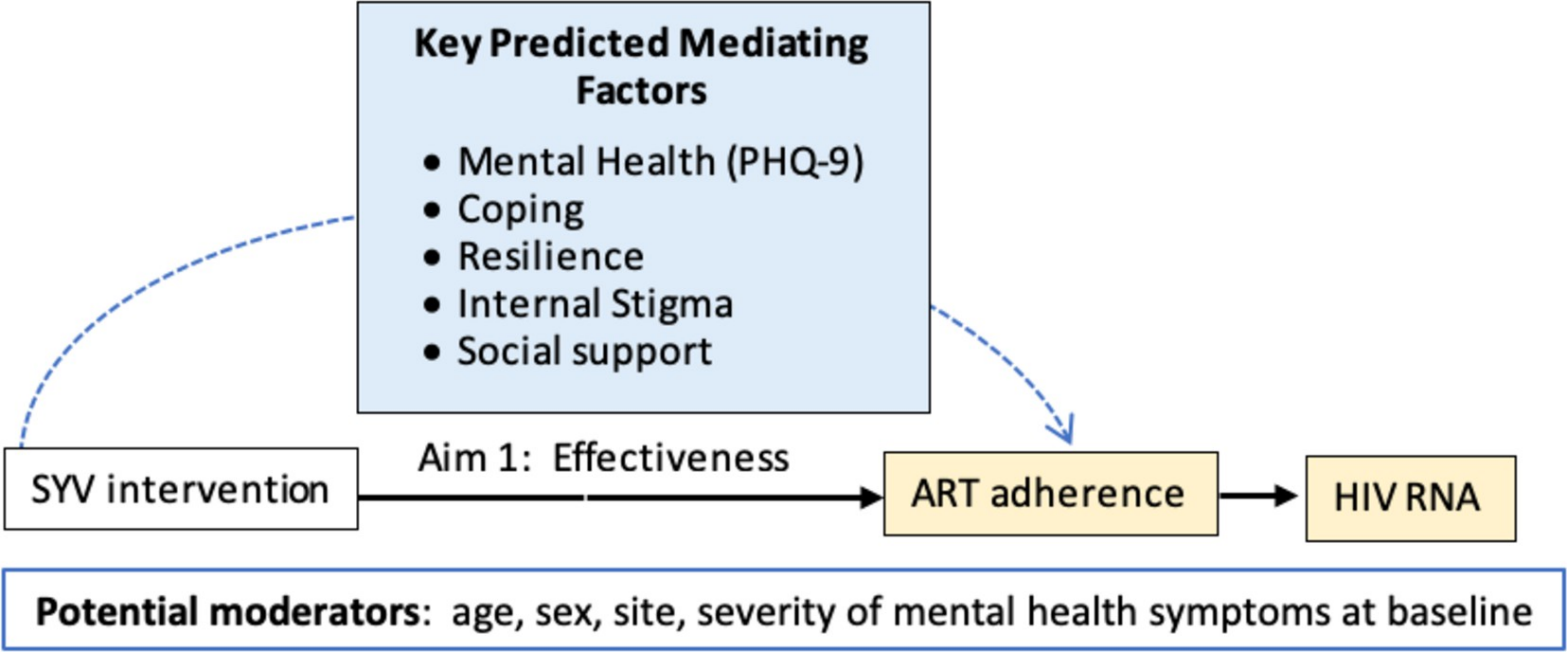
Simplified SYV intervention proposed mechanism of change.

*AIM 2 (Mechanism of change for SYV)*. The hypothesis of mediation of the intervention effect will be evaluated using the five continuous mediators—depression, coping, resilience, internal stigma, and social support (see Fig 7). To ensure the time aspect of causal mediation, the effect of intervention on the mediators at 4-months (T1) and then the effect of the mediators on the outcomes at 6 months (T2) will be examined.

*AIM 3 (Implementation outcomes of SYV) includes qualitative and economic analyses*: *Qualitative analysis*. Qualitative data will include 1) two distinct IDIs with SYV Peer Group Leaders (n = 25 participants) and focus groups with SYV participants (n = ~32 groups * 5–10 participants per group) CFIR domain: individuals involved; 2) IDI informant interviews with clinical staff from each site (n = ~10) CFIR domain: inner setting; 3) national and regional government and NGO leaders (n = ~6) CFIR domain: outer setting (See Fig 6). Prior research suggests that thematic saturation can be achieved with 12 interviews, with meta-themes presenting as early as six interviews [68]. The proposed sample size should be sufficient to achieve thematic saturation of facilitators and barriers to implementation of SYV from various stakeholder perspectives, and likely at the site level for SYV participants given that the number of FGDs needed for saturation is estimated as three to five [69]. Thematic analyses will be conducted in QSR Nvivo (or equivalent software) using a codebook made up of *a priori* and emergent structural codes based on the interview guide and four interrelated steps: reading, coding, data display, and data reduction [70].

#### Economic analysis

A cost-effectiveness (CEA) analysis is planned to assess the value-for-money of implementing the SYV program in Tanzania using established guidelines for conducting CEAs alongside clinical trials [71]. The CEA will take a program-level, health system, and societal perspective and will cover the time horizon of the patient’s involvement in the study. Two main outcomes are the focus: the number of patients virally suppressed and disability-adjusted life years (DALY’s) averted as a result of the SYV intervention. For each outcome, incremental cost-effectiveness ratios using Eq 1 will be used. Health outcome estimates will be obtained from program records. Costs will be estimated using the hybrid-costing approach [72]. Patient-level cost data will be collected using a patient cost survey conducted at each study visit to estimate direct and indirect costs (e.g., income loss due to illness and care-seeking) and any cost difference between the SYV and SOC arms. Financial reports submitted for accounting from the project will be used to estimate program-level and research costs. These include costs associated with service delivery personnel, supplies, laboratory investigations, patient medical costs, training, management, demand-generating activities, equipment, overhead (e.g., administration, indirect personnel, building, and general equipment), and patient transportation. To test the robustness of the estimates, sensitivity analysis using a bootstrap method will be conducted [73].


$$Incremental\;Cost\;Effectiveness\;Ratio=\frac{Cost_{B}-Cost_{A}}{Effect_{B}-Effect_{A}},$$ Eq 1

### Monitoring

The study has a data safety monitoring board (DSMB) consisting of a research psychologist, a social scientist, a pediatric infectious diseases specialist with expertise in adolescent HIV, and a biostatistician (all of whom work in sub-Saharan Africa). The DSMB is independent from the sponsor. Due to the nature of the study being non-invasive, the DSMB determined that stopping guidelines are not required. Interim analysis is shared quarterly with the DSMB to advise on continuation of the trial at each analysis.

Adverse (AE) and serious adverse events (SAE) are reported in the quarterly report. Any SAEs including hospital admission, suicide attempt, violence towards others, or death for any reason are reported to the DSMB and ethics committees no more than 3 working days after detection. All AE and SAE are documented in the REDCap data base including information regarding referrals for counseling, psychiatry, psychotropic mediation and whether ongoing follow up is required regardless of intervention arm.

A data quality report is generated daily and is reviewed weekly with the program manager and research assistants. No independent firm has been hired for auditing at the time of writing this protocol paper due to limitations in trial budget and auditing costs.

### Ethics

Ethical approvals were received by all IRBs including Duke University (Pro00109309), Kilimanjaro Christian Medical Centre (#2542), Ifakara Health Institute (IHI/IRB/EXT/No: 33–2023), Baylor Center of Excellence in Mbeya (SZEC-2439/R.E./V.1/27), and the Baylor Center of Excellence in Mwanza defers to the National Institute of Medical Research (NIMR/HQ/R.8c/Vol.I/2358). Data transfer and material transfer agreements are signed and executed across all sites. Any modifications to the protocol are communicated in writing to all the ethical approval bodies for approval or protocol amendment.

#### Dissemination policy

The trial is registered on ClinicalTrials.gov (NCT05374109) to make information about the study and study progress readily available. Importantly, research findings are shared with the local community through the youth and adult community advisory boards (CAB). Each SYV site works closely with a youth CAB whereby research updates are presented bi-annually. In addition, the SYV team ensures that research findings are disseminated in National meetings and conferences such as at the government sponsored quarterly adolescent technical working group; the annual social and behavioral sciences symposium held at KCMC; the annual Tanzania Pediatric Congress; and other regional and national meetings to keep government officials and implementing partners updated on research progress. All site SYV investigators are encouraged to submit abstracts and present findings at both local and international research meetings.

Authorship guidelines follow those of the ICJME [74]. The intention is to publish in open access journals and to provide access to data and statistical code once trial results are published. Data are planned for deposit in an approved data repository upon completion of the clinical trial.

### Confidentiality

Confidentiality of participant information is of prime importance in the SYV trial. Study ID numbers are used in lieu of participant names whenever possible. Study data are securely stored on technologies that are compliant with HIPAA and other local, institutional, and national policies, regulations, and laws governing sensitive data. Confidentiality forms are signed at the onset of all new study intervention waves to ensure participants and caregivers in group understand the rules of confidentiality with the mantra, “What is said in the group stays in the group”. The rule for confidentiality is also reviewed at the beginning of each group session. Exceptions to this are stipulated in the consent form and study protocol. If a participant is at risk of causing harm to self or others, confidentiality is broken for the purposes of safety.

## Discussion

YPLWH are at the center of the HIV epidemic in sub-Saharan Africa. The SYV clinical trial protocol outlines a RCT designed for rigorous evaluation of the effectiveness and implementation of the SYV intervention to address the mental health challenges in adolescent HIV care in Tanzania. SYV was developed as a manualized, group-based, peer-led intervention and utilized youth participatory methods to incorporate common stressors and worries faced by YLWH with particular attention to stigma, disclosure, and social support (Fig 4) [21]. At the time of the intervention development, there was no mental health department, nor psychologist or psychiatrist in the Kilimanjaro region [26]. The intervention relied heavily on social workers and trained nurses. As the trial is aiming to scale in 2023/2024, attention to site differences across the four regions and level of mental health care available for referral are carefully monitored to evaluate any associated implementation and research outcome differences across sites. Few prior studies have identified effective, systematic mental health referral systems for adolescents engaged in research in low resource settings. Ensuring participant safety and establishing best practice for adolescent mental health referral across a variety of contexts in Tanzania will significantly contribute to best practice guidance in handling mental distress among adolescents who participate in research studies and inform new referral systems as part of SOC.

Due to limited mental health resources in Tanzania, task-sharing strategies are used to deliver the SYV intervention. Twenty-five PGL are trained in SYV delivery. During training, sessions 4 through 6 (Fig 4) that unpack the HIV disclosure trauma narrative were noted to be particularly emotional for the PGL. Many group leaders still carried their own intense trauma from when they learned they live with HIV. Site supervisors and mental health specialists at training supported the PGLs as they processed their own histories. This was a critical step for PGLs to come to accept their own narrative before they could support study participants.

The trauma narrative is a unique and critical component of SYV that was adapted from TI-CBT [36] and prior work with Tanzanian children recently orphaned and coping with grief [34]. PGLs have since participated in multiple training sessions regarding “secondary or vicarious trauma,” the trauma-related symptoms they may experience when the story of a participant is very similar to their own. In response, group leaders form their own support system using WhatsApp groups. They continue support each other across sites along with support from their local site supervisors and principal investigators.

Integrating mental health in the care of YPLWH is critically important [75]. Other peer-led interventions are currently being evaluated to fill this mental health gap [76]. Bright Futures, originally piloted in Rwanda [77], is a peer-led, six-session, group-based intervention enrolling youth with moderate to severe mental distress with the primary outcome being change in mental health (IMPAACT 2016) [78]. Zvandiri, a program from Zimbabwe utilizes Community Adolescents Treatment Supporters or “CATS” who are YPLWH between 18–24 years of age. CATS act as community liaisons to improve adolescents’ HIV treatment outcomes, sexual and reproductive health, and mental health [79]. Zvandiri-CATS partnered with the Friendship bench, a problem-solving mental health intervention from Zimbabwe [80], to address mental health challenges among YPLWH. The randomized trial did not show a difference on the primary outcome of viral suppression, but it did show improved mental health outcomes, with greater improvement noted in the problem-solving Friendship Bench arm than routine CATS [81]. Important qualitative work with the CATS informed the TRUST framework, a practical guide to providing supportive infrastructure and supervision for peer youth leaders [82].

As more evidence becomes available, it is important to consider scale and sustainability of these interventions. SYV uniquely incorporates a mechanism of change analysis that will help identify the most critical sessions and understand for whom the intervention works best. A cost-analysis is expected to provide important cost implications to inform government and policy makers on cost-effectiveness and strategies to best incorporate the intervention into routine clinical care.

The SYV trial is not without potential limitations. First, there is inherent risk of contamination of intervention content being shared between participants randomized to receive SYV with those randomized to SOC due to the intra-clinical site randomization. This may make it difficult to observe the difference in outcomes between the SYV intervention and SOC group. Many of the group leaders have second jobs as a peer navigators or in clinical home based care services within the adolescent HIV clinic. They could inadvertently share the information they teach in SYV with youth who are in the control group in need of their SOC service. To mitigate this risk, all participants are asked “contamination questions” to understand their level of exposure to unique SYV content. Second, given the increasingly robust ART treatment regimens, virologic suppression has fortunately improved across adolescent HIV clinics in Tanzania. Most study sites report that greater than 80% of youth attending adolescent HIV clinic are now fully suppressed. Study research assistants work hard to identify youth with virologic failure based on their SOC viral load (HIV RNA ≥400 copies/mL) and to enroll them into the study. Third, the referral system developed to address moderate to severe mental distress and safety concerns in all participants regardless of trial arm are beyond that of usual standard of care. This enhanced mental health care for YPLWH could weaken the ability to demonstrate the effect of SYV. Finally, training and supervision of PGL is time intensive. PGL attrition could limit group session delivery. In the original pilot there was no attrition and all original PGL have continued on the current project as “expert group leaders”. This experience makes PGL attrition in the current trial seem less concerning. Despite these potential limitations, the SYV trial has tremendous potential to further improve HIV outcomes and the mental health trajectory for YPLWH.

## Supporting information

S1 Checklist SPIRIT 2013 checklist: Recommended items to address in a clinical trial protocol and related documents*. (DOC)

S1 File (PDF)
