# Supplementary material for: Study protocol for Sauti ya Vijana (The Voice of Youth): A hybrid-type 1 randomized trial to evaluate effectiveness and implementation of a mental health and life skills intervention to improve health outcomes for Tanzanian youth living with HIV
Source: PLoS One. 2024 Aug 26;19(8):e0305471. doi: 10.1371/journal.pone.0305471 (PMC11346953; doi:10.1371/journal.pone.0305471)
Supplement: S1 File — (PDF) [file pone.0305471.s002.pdf]

# SYV: A Mental Health Intervention to Improve HIV Outcomes in Tanzanian Youth

**Version 2, July 28, 2022**

Co-Principal Investigator
**Dorothy Dow, MD, Msc-GH**
Pediatric Infectious Diseases,
Duke University and

Co-Principal Investigator:
**Blandina Mmbaga, M.D., PhD**
Department of Paediatrics
Kilimanjaro Christian Medical Centre

Co-Principal Investigator:
**Getrud Mollel, M.D., MSc**
Chronic Disease Clinic of IfakaraIfakara Health Institute

Co-Principal Investigator:
**Jason Bacha, M.D.**
Baylor College of Medicine Children's Foundation – Tanzania
Mbeya Centre of Excellence

Co-Principal Investigator:
**Judith Gwimile, MMED**
Baylor College of Medicine Children's Foundation – Tanzania
Mwanza Centre of Excellence

Co-Investigator:
**Aisa Shayo, MMED**
Department of Paediatrics
Kilimanjaro Christian Medical Centre

Co-Investigator
**Judith Boshe, MMED**
Department of Psychiatry
Kilimanjaro Christian Medical Centre

Co-Investigator
**Bernard Desderius MD, MMED, FPC, MPH**
Head, HIV &TB Programs
Bugando Medical Centre, Mwanza Tanzania

**SYV: A Mental Health Intervention to Improve HIV Outcomes in Tanzanian Youth**,
Version 2: July 28, 2022

**Table of Contents**


ABBREVIATIONS 3
ABSTRACT 4
INTRODUCTION 5
LITERATURE REVIEW 6
THE PROPOSED INTERVENTION 7
PROBLEM STATEMENT 10
RATIONALE 10
BROAD OBJECTIVE 11
SPECIFIC OBJECTIVES/AIMS 11
METHODOLOGY 11
STUDY AREA 11
INTERVENTION TRAINING AND SUPERVISION 12
STUDY DESIGN 13
STUDY POPULATION 14
INCLUSION AND EXCLUSION CRITERIA: 14
SAMPLE SIZE CALCULATION 14
*NUMBER RECRUITED AND RANDOMIZED 15
SAMPLING TECHNIQUE/RECRUITMENT 15
DATA COLLECTION AND TOOLS 17
RANDOMIZATION 19
STUDY VARIABLES AND OUTCOMES 20
LABORATORY CONSIDERATIONS 21
DATA MANAGEMENT ERROR! BOOKMARK NOT DEFINED.
ETHICAL CONSIDERATIONS 23
INSTITUTIONAL ETHICAL CLEARANCE 23
CONSENT: 24
LIMITATIONS OF THE STUDY 25
INVESTIGATOR ROLES 25
DISSEMINATION OF THE RESULTS 26
DETAILED BUDGET AND BUDGET JUSTIFICATION 26
SUMMARY TABLE OF BUDGET PER SITE* 26
BUDGET JUSTIFICATIONS: 27
REFERENCES 30

ABBREVIATIONS

ART: Antiretroviral Therapy
CAB: Community Advisory Board
CBT: Cognitive Behavioral Therapy
FDG: Focus Discussion Group
HIV: Human Immunodeficiency Virus
IDI: In-Depth Interviews
IPT: Interpersonal Psychotherapy
KCMC: Kilimanjaro Christian Medical Centre
MI: Motivational Interviewing
NIMR: National Institute Medical Review
RCT: Randomized Controlled Trial
SAT: Social Action Theory
SOC: Standard of Care
SYV: Sauti ya Vijana (The Voice of Youth intervention)
TI-CBT: Trauma-Informed Cognitive Behavioral Therapy
U=U: Undetectable = Untransmitable
YPLWH: Young People Living with HIV

## ABSTRACT

**Background:** Young people living with HIV (YPLWH, 10-24 years of age) are a growing population that experience unique mental health challenges that may compromise their HIV care. Despite the clear need, few evidence-based mental health interventions exist to address the difficulties faced by this important population.

**Methods:** The trial will start with a small pilot to ensure feasibility and smooth logistics operations followed by a randomized controlled trial across four sites in Tanzania. Data for quantitative analysis will be collected via: a structured questionnaire evaluating demographics, behaviors, stigma, adherence, and mental health; medical chart abstraction to obtain data on primary outcome measures; and obtaining biologic specimens (hair sample and blood sample) for measures of adherence and viral load. Study visits will occur at baseline/enrollment, and post-intervention, 6-mo, 12-mo and 18-mo post-enrollment with a window period around the study assessment. Additionally, indepth interviews will be conducted among key stakeholders including both the inner setting (health care providers and clinic staff, group leaders that deliver the intervention and supervisors of the intervention) as well as the outer setting (policy makers in the ministry of health, participants of the intervention, caregivers, and other key stakeholders).

**Results**: The trial will report on change in key outcomes including but not limited to mental health, adherence, viral load, cost, and feasibility and acceptability of the intervention at each site.

**Conclusion:** This project is expected to fill a critical mental health gap in caring for YPLWH in Tanzania with far-reaching implications on individual health (HIV suppression) and population health (preventing transmission of HIV infection to others).

**Summary Statement:** Young people living with HIV (YPLWH, 10-24 years of age) are a growing population that experience unique mental health challenges that may compromise their HIV care. Despite the clear need, few evidence-based mental health interventions exist to address the difficulties faced by this important population. The *goal* of our research is to provide a developmentally appropriate, evidence-based mental health intervention that effectively helps YPLWH cope with life challenges, instill hope for the future, and find motivation to effectively take their antiretroviral therapy medication leading to improved HIV outcomes. *Sauti ya Vijana (SYV, The Voice of Youth), is a novel and innovative group-based mental health and life skills intervention* designed with and for Tanzanian YPLWH to address the mental health and life challenges they have described in our prior research. The intervention consists of 10 group sessions (two joint sessions with caregivers or a supportive adult) and two individual sessions delivered by trained young adults living with HIV who have successfully transitioned to the adult clinic using a task sharing model that builds local capacity while overcoming the critical shortage of mental health professionals in this setting. SYV incorporates components of Trauma Informed-Cognitive Behavioral Therapy,

Interpersonal Psychotherapy, and Motivational Interviewing that during this critical neurodevelopmental period, when foundations of self and social regulation are realized, may help to prevent or dramatically reduce severity of mental health symptoms. We have piloted the SYV intervention in Moshi, Tanzania and the data from the pilot showed that SYV was acceptable, feasible, and estimated a 10% improvement in virologic suppression for those who received the intervention compared to those who did not. The *overall objectives* of this proposal are to support positive coping strategies that bolster mental health and lead to improved HIV outcomes among YPLWH. The *central hypothesis* is that SYV will be effective to improve antiretroviral therapy (ART) adherence and virologic suppression in YPLWH in Tanzania. The *rationale* for this project is that by targeting mental health, which is strongly associated with medication adherence, we will effectively improve adherence and thereby HIV viral suppression. The central hypothesis will be tested in three aims in a hybrid type-1 effectiveness-implementation trial. The first aim will first pilot the intervention across four key regions including Kilimanjaro, Mwanza, Mbeya, and Ifakara to ensure the logistics of the study design are feasible and acceptable. Then, a fully powered individually randomized group treatment trial will determine if SYV is effective based on the primary outcome of a 10% increase in virologic suppression (HIV RNA <400 copies/mL) among YPLWH in the intervention arm compared to standard of care. The second aim will elucidate the mechanisms by which SYV works and for whom it is most effective by evaluating potential moderators and mediators of the intervention using structural equation modelling. Change in mental health, internal stigma, resilience and coping are hypothesized to mediate the intervention effect, while age, sex, site, and baseline mental health symptoms are hypothesized moderators. The third aim will evaluate potential barriers and facilitators to SYV trial implementation, including cost effectiveness and sustainability using the Consolidated Framework for Implementation Research and mixed methods outcome measures. This project is *significant* because it is expected to fill a critical mental health gap in caring for YPLWH in low resource settings with far-reaching implications on individual health (HIV suppression) and population health (preventing transmission of HIV infection to others).

## INTRODUCTION

**Reduction in AIDS related mortality has not been realized in young people living with HIV (YPLWH) at the same rate as other age groups.** An unprecedented 1.8 billion young people (10-24 years of age) are alive today[1,2]. In Africa, 60% of the continent's population is under the age of 25 years, and this is where a staggering 85% of the world's YPLWH reside[3]. An estimated 1600 new HIV infections occur every day in youth 15-24 years of age, and a young person dies every 10 minutes due to AIDS-related illness[4], far more than any other age group. YPLWH are a unique and vulnerable population who harbor a high burden of global HIV infection and are a major contributor of HIV transmission. Innovative interventions tailored to their specific needs are urgently needed to combat these devastating outcomes and prevent the spread of HIV.

**The added burden of living with HIV during the developmental period of adolescence**

**can be devastating.** For young people in general, hormonal and physical changes, coupled with ongoing brain development, partially explain their tendency for risk taking, substance use, and the emergence of psychopathology[5]. In addition to dramatic biologic changes, their social environments are rapidly changing. Peer networks and "fitting in" become priority and can compromise rational decision making[6,7]. YPLWH have the added burden of navigating peer and romantic relationships while living with a stigmatizing, sexually transmittable infection that requires adherence to daily treatment. These factors contribute to worse HIV outcomes among young people compared to children or adults[8-10], largely related to delayed diagnosis, loss to follow-up, and inadequate adherence to antiretroviral therapy (ART)[11,12]. A key factor associated with inadequate ART adherence is mental health difficulties that often emerge during adolescence[13-18].

**Despite the established role of mental health in ART adherence, in Africa there is a striking lack of resources devoted to mental health[19,20].** Failure to address the mental health gap in the vulnerable and growing population of YPLWH threatens progress towards ending HIV/AIDS as a public health threat by 2030[21]. We know that virologic suppression (undetectable) prevents ongoing HIV transmission (untransmittable),[22] or "U=U"[23]. YPLWH represent a known and specific obstacle to realizing this goal given their risk for low ART adherence, active HIV replication, and emerging sexual activity. It is estimated that only 50% of YPLWH in Africa are virologically suppressed[24]. Reaching the remaining 50% will be exponentially harder than achieving suppression in the first 50% and new innovations towards this goal are urgently needed. In response, we propose a highly promising mental health and life skills intervention that engages YPLWH and their caregivers in group-based therapy designed to help navigate these unique challenges, emphasizing excellent ART adherence.

## LITERATURE REVIEW

**Mental health interventions effectively improve ART adherence in high income countries[25,26]and show proof of concept in adults in low resource settings[27-29]; however, evidence-based interventions designed to address the unique mental health challenges faced by YPLWH in low resource settings are lacking[30,31].** A family-based psychosocial intervention developed in the United States, CHAMP+[32], was adapted and piloted in South Africa and Thailand with promising results for young adolescents (10-14 years of age) living with HIV and their families[33,34]. Unfortunately, the intervention does not address the 5 million older adolescents and emerging adults (15-24 years of age) currently living with HIV who are at greatest risk for ART non-adherence and HIV transmission through sexual activity[35]. The Zvandiri Community Adolescent Treatment Supporters (CATS) intervention in Zimbabwe, although promising, does not specifically address mental health and contains no specific scripted content for quality assurance nor fidelity[36,37]. In Rwanda, the Kigali Imbereheza Project (KIP) used a Trauma-Informed Cognitive Behavioral Therapy (TI-CBT) peer youth delivery model, but outcomes are unpublished[38]. An efficacy trial based on KIP (IMPAACT 2016) has the primary outcome of improving mental health, not HIV outcomes, and is not yet enrolling[39]. There remains a critical gap in evidence-based mental health interventions to meet the specific needs of YPLWH in Africa, where the true burden of disease lies.

**Understanding the mechanisms by which mental health interventions influence biologic outcomes and for whom they work best is critically important, yet not fully known.** During adolescence, neurocognitive and pubertal maturation interact with the social determinants of health, creating a highly dynamic health profile[40]. Multiple variables are interconnected with complex relationships that may influence the development of psychopathology that ultimately influence HIV health outcomes.[18,41,42]. Multiple studies in YPLWH in low resource settings document the importance of psychosocial factors (stigma, coping, resilience and mental health) that influence ART adherence[43,44], but the pathways by which they exert this influence are complex and poorly understood. A deeper understanding of these mechanisms as they relate to interventions for YPLWH are needed to understand *how* and *for whom* they work best.

**Of the few mental health interventions tailored for children and adolescents in low resource settings, fewer still have moved beyond the pilot stage to establish effectiveness and evaluate implementation at scale.** A recent systematic review explored determinants of implementing evidence-based trauma-focused interventions for children and youth using the EPIS (Exploration Preparation Implementation Sustainment) framework[45]. Two studies in Africa evaluated participant and counselor perspectives on TI-CBT delivery for bereaved children, one in Zambia,[46] the second in Tanzanian and Kenya.[47] Implementation outcomes demonstrated interventions were appropriate and acceptable, but did not evaluate implementation outcomes of cost or sustainability. Similarly, the TI-CBT intervention, KIP, for YPLWH in Rwanda, retrospectively applied the EPIS framework, but implementation outcomes were also limited to acceptability, feasibility, uptake, and fidelity[48]. The Friendship Bench, an evidenced-based problem solving mental health intervention delivered individually to adults (>18 years) in 6-12 weekly sessions by lay counselor "grandmothers", is a good example of an intensive mental health intervention that has been sustained and brought to scale. After promising pilot data[49], demonstrating effectiveness[50] and engagement with key stakeholders[51], the intervention has been successfully scaled to 72 clinics in Zimbabwe[52]. The complex Zvandiri CATS intervention has similarly been scaled to 613 clinics despite costing data that showed it cost three times that of standard of care[37]. Cost-effectivenessness analysis of CATS is underway[37]. Though scale up and sustainability of evidence-based mental health interventions is challenging, examples from Zimbabwe show that incorporating task-sharing delivery and close collaboration with key political stakeholders may be a successful strategy to overcome barriers of scale and sustainability[53].Few interventions for YPLWH in Africa have evaluated important implementation outcomes of cost and sustainability, but this information is critical to inform policy-makers' investment in sustaining and not shelving effective interventions.

## THE PROPOSED INTERVENTION

**Our team developed a mental health and life skills intervention, Sauti ya Vijana (SYV, The Voice of Youth) specifically tailored to the needs of YPLWH in Tanzania.** Building on the well-established finding that mental health is associated with ART adherence and HIV outcomes[54], we designed SYV to address the specific challenges YPLWH in Tanzania

described in our formative research[55,56]. We demonstrated that in Tanzania, 32% of YPLWH reported symptoms of mental health difficulties (depression, post-traumatic stress, and/or emotional/behavioral problems) that were significantly associated with how participants learned that they live with HIV (primary HIV disclosure), stigma, and worse self-reported adherence[55,57]. To reach a large number of youth while building on the communal aspect of Tanzanian culture and the importance of peers, we designed SYV as a group-based mental health and life skills intervention. SYV includes 10 group sessions (two sessions held jointly with caregivers) lasting approximately 90 minutes and two individual sessions delivered by trained young adult group leaders who use a manualized protocol that is designed to scale in low resource settings. The intervention is applied to the Social Action Theory (SAT), a theoretical framework used to determine factors that influence health behavior[58]. Building off a SAT resilience framework for YPLWH[59], we strategically use components of evidence-based treatment models to influence cognitive, self, and social regulation to improve behavioral health outcomes **(Figure 1)**. SYV integrates components of three psychotherapies: TI-CBT[60], which in the developing brain is thought to promote cognitive restructuring and influence connectivity from the amygdala towards the prefrontal cortex, an area associated with planned behavior and reasoned decision-making[61]; Interpersonal Psychotherapy (IPT)[62], which is designed to improve social relationships, especially as support for traumatic experiences and life transitions; and Motivational Interviewing[63] (MI) along with value-guided goal pursuit[64]. During this pivotal developmental period when critical foundations of self and social regulation are realized, SYV may help prevent or reduce the severity of psychopathology, thereby improving health outcomes[65].

**SYV content is designed to address the stress and trauma experienced by YPLWH (see Figure 1 below).** SYV is based on a client-centered approach, designed to provide an empathic and educational environment to promote self-efficacy and resilience without judgement or confrontation[66]. Caregivers (supportive adults) are invited at the discretion of enrolled youth to participate in two sessions (session 1 and session 6).

These sessions include both time with caregivers separate from youth to explain the intervention content and communication strategies, and time together with youth to further facilitate communication and emotional support for youth.

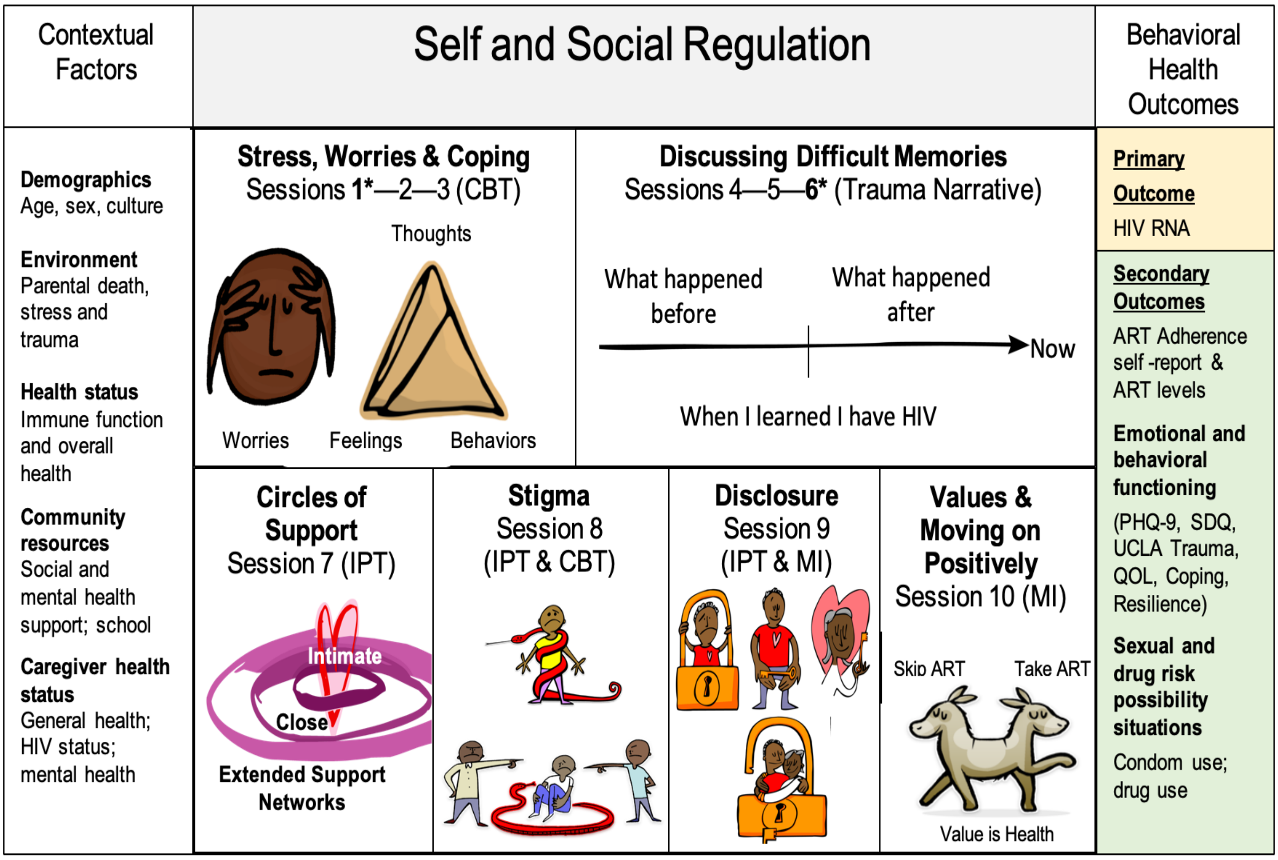


Figure 1: Sauti ya Vijana (SYV) Applied to the Modified Social Action Theory Framework[58,75]

Abbreviations: CBT: Cognitive Behavioral Therapy; IPT: Interpersonal Psychotherapy: MI: Motivational Interviewing; ART: antiretroviral therapy; PHQ: Patient Health Questionnaire; SDQ: Strengths and Difficulties Questionnaire; QOL: quality of life

*Two sessions (one and six) are joint with caregivers; Not shown are two individual sessions (after session 4 and after session 10)

In the first three sessions, youth participants name their worries, discuss coping strategies, practice relaxation and breathing exercises, and learn components of CBT (how one's learned patterns of thinking often influence feelings and behavior). The Trauma Narrative, derived from CBT[67], uniquely addresses the experience of when young people learned they live with HIV. In our formative work, we learned that the young person's primary HIV disclosure was frequently linked to a traumatic memory such as parental death or change in living environment[56]. To help participants come to terms with the trauma, sessions four through six are dedicated to reflection and processing the experience. A timeline derived from IPT is used to help youth discuss these difficult memories individually with a group leader, with peers in a group session, and with caregivers in a supportive environment. IPT is the crux of session seven whereby youth name their support network and identify any desired shifts in those relationships. Components of IPT and CBT continue in sessions eight and nine as participants consider stigma (illustrated as a snake) in their lives and to whom they may (or may not) consider disclosing their HIV status (illustrated as giving someone the key to unlock their innermost secret). Paired with session nine are discussions regarding sexual reproductive health, condom use, and gender-based violence. Sessions nine and ten expand the overall

client-centered approach to MI principles emphasizing autonomy rather than imposing ideas about what the youth "should" do. MI principles help participants make independent choices (illustrated as the "push/pull" of a two-headed donkey with their value at the core) compatible with their values and future goals, acknowledging that with ART adherence they can live long and healthy lives. In a final individual meeting, participants revisit their personal values, goals, and strategies for the next six months and review their support networks as SYVcomes to end. A final gathering is used to review all session content, to celebrate all that has been shared and learned together, and to distribute certificates of completion.

## PROBLEM STATEMENT

Young people living with HIV (YPLWH, 10-24 years of age) are a growing population that experience unique mental health challenges that may compromise their HIV care. Despite the clear need, few evidence-based mental health interventions exist to address the difficulties faced by this important population. Every 10 minutes a young person 10-24 years of age dies of an AIDS-related illness. The high mortality in this age group is largely due mental health challenge associated with incomplete adherence to antiretroviral therapy (ART). This study proposes to address the mental health care gap for YPLWH in Tanzania.

## RATIONALE

In our pilot study conducted in Moshi, Tanzania we found that the SYV intervention was feasible, acceptable, and demonstrated promise to improve mental health, ART adherence, and virologic suppression. **Evaluating SYV in a fully-powered effectiveness-implementation (hybrid type-1) trial will substantially contribute to the field by providing the first developmentally tailored mental health intervention for YPLWH powered to show change in viral suppression in Tanzania.** This study will provide a unique and rigorously tested, evidence-based, mental health and life skills intervention developed for YPLWH in an African context that is manualized to ensure fidelity and scalability. YPLWH will benefit from the intervention with improved skills for coping with HIV, improved adherence to medication and therefore suppression of the HIV virus. Group leaders who are trained and supervised to deliver the intervention will benefit in gaining knowledge about mental health, leadership and resilience to be leaders in their community. Supervisors of the intervention will gain further skills in supervision and mental health interventions as well as gaining knowledge about HIV. The adolescent HIV clinic will benefit by bringing a way to address the pervasive mental health challenges faced by these youth and data to show cost-effectiveness towards integrating the intervention into the clinic in a sustainable way. The community will benefit from the intervention by an increase in mental health awareness and more YPLWH having HIV suppression, thus less likely to transmit HIV in the community. The country will benefit from this trial as it is positioned to contribute important scientific knowledge that will advance the adolescent mental health and HIV field and improve health outcomes for YPLWH in Tanzania and across the African continent.

## Broad Objective

To improve mental health, HIV care, and virologic outcomes among YPLWH by addressing the mental health gap in HIV care in Tanzania.

## Specific Objectives/Aims

**1.** Determine the effectiveness of SYV versus standard of care to improve ART adherence and virologic outcomes in Tanzanian YPLWH in an individually randomized group treatment-controlled trial.
**2.** Elucidate the mechanisms of change to explore how and for whom the SYV intervention is most effective.
**3.** Evaluate implementation outcomes, including cost-effectiveness, of the SYV intervention guided by the Consolidated Framework for Implementation Research (CFIR).

## METHODOLOGY

The proposed study is based on the original pilot study of SYV conducted in Moshi, Tanzania. Results from the original pilot trial demonstrated that the intervention was feasible, acceptable, and may lead to improved ART adherence and virologic outcomes for YPLWH. In this protocol we propose to scale the intervention to include Moshi and additionally sites in Ifakara, Mbeya, and Mwanza region. The approach is to first pilot the study at all sites to practice the logistics of study visits and running the intervention. After any needed adaptations are implemented to improve trial logistics, we will then perform the randomized controlled trial to evaluate the effectiveness of SYV.

## Study Area

The East African country of Tanzania is home to approximately 5% of the world's adolescents living with HIV (98,000 adolescents, 10-19 years of age)[68]. The country is large and diverse, four times the size of Uganda, and although Swahili is the official language, over 100 distinct tribes make up the population of over 57 million people[69]. In 2018, the estimated HIV prevalence was 4.6%[70]. Among YPLWH in Tanzania, 42% were virologically suppressed[71].The sites for the proposed study (with HIV prevalence of the region in parentheses) are Mbeya (9.3%), Mwanza (7.2%), Ifakara (4.3%), and Moshi (2.6%)[72]. These four sites were purposefully chosen based on: **1)** completion of a site survey demonstrating interest in participation, **2)** having an established adolescent HIV clinic, **3)** perceived mental health need identified by clinic staff, **4)** serving a large population of >200 YPLWH, **5)** 20% or greater virologic failure rates, and **6)** demonstrated research infrastructure (suitable venue to conduct the intervention, site personnel trained in research and able to act as a local site supervisor, and ability to obtain, test, and store HIV RNA samples). In all sites, ART and standard of care HIV-1 RNA (viral load) are available free of charge. We recognize the majority of participating sites operate within tertiary care centers and many YPLWH will access care at lower level facilities. In keeping with a hybrid type-1 trial design, we prioritized sites with research infrastructure to robustly and reproducibly demonstrate intervention effectiveness in four different regions of the country prior to focusing on implementation at different levels of facility care.

## Intervention Training and Supervision

Each trial site will designate a study supervisor with experience in counseling YPLWH. The study supervisor and site PI will interview and hire four to six young adult group leaders (half female/half male). Young adult group leaders will be 23-29 years of age upon hiring, live with HIV, demonstrate excellent ART adherence, have documented viral suppression (HIV RNA <400 copies/mL) for at least 6 months, exhibit emulative behavior (no current challenges with alcohol/drugs based on clinical staff, references, interview), and ideally have experience as a peer leader or mentor. Study activities are expected to occur primarily in Kiswahili. Once each site identifies this team, all study site PIs, supervisors, and group leaders will attend a two-week intensive SYV training in Moshi, Tanzania as was done for the original pilot study. The training will be led by the Tanzanian psychiatrist, Dr. Boshe, with support from the intervention developers (Dr. O'Donnell and Dr. Dow) explaining the intervention theory and modeling session delivery with help from the six Moshi-based "expert" SYV group leaders who delivered SYV during the original pilot. Afternoon sessions will utilize a cascading model of supervision[73] (**Figure 2**) with site specific break-out sessions to practice the intervention material in small team groups. Breakout sessions will be supervised by two to three experienced Moshi team members [one expert supervisor (study one or one or two expert group leaders] per field site.

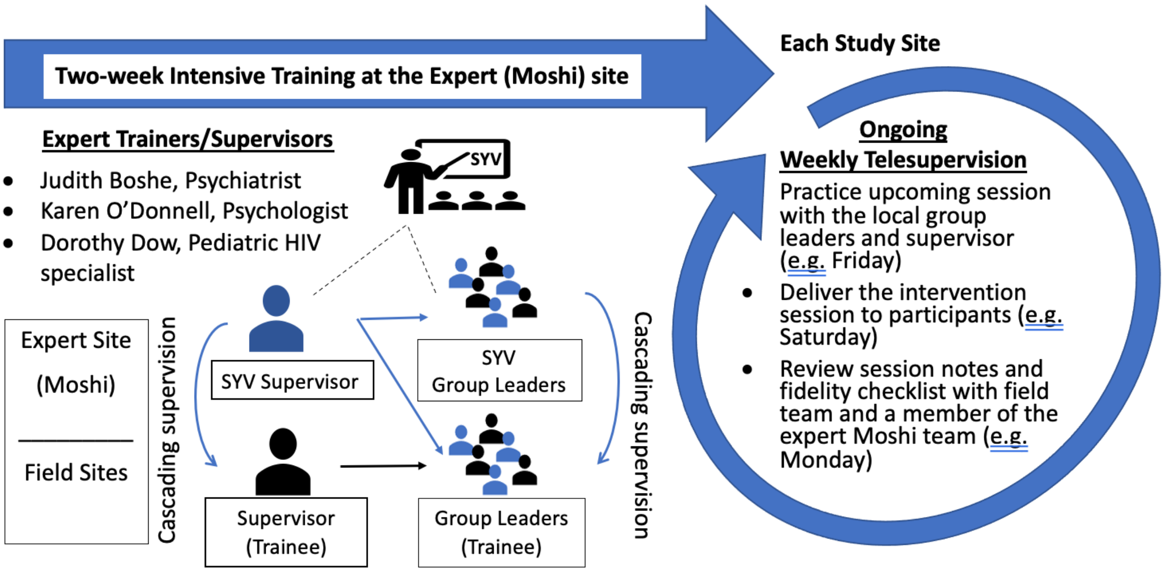


Figure 2: Capacity building using the cascading model of supervision during the two-week intensive training and ongoing weekly practice and telesupervision at each study site

At the end of the two-week intensive training, group leaders at all sites will continue to practice the concepts and intervention content at their home site with ongoing training and supervision from the local supervisor. At each site, group leaders will practice all intervention sessions at least twice prior to delivery to live participants. The intervention session practice will be with two group leaders who will will deliver the session to an audience of the other four group leaders plus the supervisor who will act as mock participants. Practice sessions may take place with several sessions back to back and several days a week in order to review session content efficiently. Group leaders should all be comfortable with the material prior to starting

the pilot. Practice of the upcoming session will continue to occur within three days of "real" group session delivery for both the pilot and the RCT.

Once the pilot and subsequently the RCT begin, each site will have a weekly, one-hour long supervision meeting. The site group leaders will discuss with their supervisor their session notes and experience during the delivered intervention session. The same expert-trainer Moshi team (an expert group leader and supervisor from the original Moshi pilot SYV program) will join the weekly site supervision call for weekly telesupervision. Supervision is a critical component of the intervention and will continue for the duration of the pilot and RCT.

All supervisors, expert trainers, and co-PIs will meet monthly via zoom to debrief on intervention delivery and any site challenges. This will help ensure fidelity and session quality are maintained for the duration of the study. This cascading model of supervision has been used successfully in other interventions in Africa[74] and ensures capacity to scale to new sites in Tanzania and other low-resource settings.

**Study Design**

The rigorous experimental design includes a pilot study followed by a parallel-arm RCT. The pilot will include approximately eight consenting members of the youth community advisory board (CAB) at each site location prior to the RCT. If a youth CAB does not currently exist at each site, one will be formed. Although we have strong pilot data for the SYV intervention from Moshi, the pilot test proposed herein will help ensure recruitment, enrollment, intervention delivery, supervision, measurement technology and logistics run as expected at each site (Moshi, Mbeya, Mwanza, Ifakara). The pilot study will be comprised of 8 participants recruited at each site. Subsequently, for the RCT, we will individually randomize up to 750 participants (see sample size calculation below while also accounting for potential participant dropout) to receive the SYV intervention or SOC to achieve 90% power to detect a 10 percentage point difference between arms in the primary outcome of virologic suppression at the two-tailed 5% significance level, accounting for clustering by SYV group in the intervention arm.

The intervention will be rolled out across four main sites, in four different Tanzanian regions, and in four waves separated in time by 6 months. There will be two SYV groups per wave at each site (~8 groups per site) (**see Table 1 and Table 3 below**). A study visit for all participants will be conducted baseline and approximately 4 months ($T_1$), 6-, 12-, and 18-months post-baseline ($T_2$, $T_3$, $T_4$) with 6-months post baseline being the primary endpoint. At 12 months post-baseline ($T_3$), those randomized to the SYV intervention will receive a one-session SYV booster to improve content retention and study engagement as has been shown in prior studies[75,76].

Table 1: Study Timeline

| | | Preparation: Training and Pilot | | | | Randomized Controlled Trial | | | | | | | | | | | | | | | | | | Wrap Up | |
|---|---|---|---|---|---|---|---|---|---|---|---|---|---|---|---|---|---|---|---|---|---|---|---|---|---|
| | | **Year 1** | | | | **Year 2 (Jan 2022)** | | | | | | | | **Year 3 (Jan 2023)** | | | | | | | **Year 4** | | | **Year 5** | |
| Month | | 1-3 | 4-6 | 7-9 | 10-12 | 1-3 | 4 | 5 | 6-8 | 9 | 10 | 11 | 12 | 1-3 | 4 | 5 | 6 | 7-10 | 11 | 12 | 1-5 | 6 | 7-12 | 1-6 | 7-12 |
| **AIM 1*** | | | | | | | | | | | | | | | | | | | | | | | | | |
| Implementation and Analyses | | Training | | Pilot **SYV** | Adapt; Recruit | Data Cleaning & Analyses (primary endpoint at $T_2$; secondary analyses; exploratory analyses) | | | | | | | | | | | | | | | | | | | |
| Wave* 1 | SYV | | | | $T_0$ | **SYV** | $T_1$ | | $T_2$ | | | | $T_3$* | | | | $T_4$ | | | | | | | | |
| | SOC | | | | $T_0$ | | $T_1$ | | $T_2$ | | | | $T_3$ | | | | $T_4$ | | | | | | | | |
| Wave 2 | SYV | | | | | | | $T_0$ | **SYV** | $T_1$ | | $T_2$ | | | | $T_3$* | | | $T_4$ | | | | | | |
| | SOC | | | | | | | $T_0$ | | $T_1$ | | $T_2$ | | | | $T_3$ | | | $T_4$ | | | | | | |
| Wave 3 | SYV | | | | | | | | | | | | $T_0$ | **SYV** | $T_1$ | | $T_2$ | | | $T_3$* | | $T_4$ | | | |
| | SOC | | | | | | | | | | | | $T_0$ | | $T_1$ | | $T_2$ | | | $T_3$ | | $T_4$ | | | |
| Wave 4 | SYV | | | | | | | | | | | | | | | $T_0$ | **SYV** | $T_1$ | | $T_2$ | | | $T_3$* | $T_4$ | |
| | SOC | | | | | | | | | | | | | | | $T_0$ | | $T_1$ | | $T_2$ | | | $T_3$ | $T_4$ | |
| **Aim 3**** (See Section C.7, Qualitative Implementation Outcome Measures and Table 4) | | | | | | | | | | | | | | | | | | | | | | | | | |
| Characteristics of the Intervention | | | | | Costing and cost effectiveness analysis; monitoring adaptations using FRAME will be ongoing from end of Y1 to Y4. | | | | | | | | | | | | | | | | | | | Ongoing Analysis Y5 | |
| Outer Setting | | | | | IDI (N=5) | | | | | | | | | | | | | | | | | IDI (N=5) | | | |
| Inner Setting | | | | | IDI (N=4) | | | | | | | | | | | | | | | | | IDI (N=4) | | | |
| Key Individuals | | | | | | | FDG, site 1 | | | FDG, site 2 | | | | | FDG, site 3 | | | FDG, site 4 | | | | IDI (N=4) | | | |
| Process | | | | | | Monitoring fidelity throughout intervention delivery | | | | | | | | | | | | | | | | | | | |

*In Aim 1, each wave represents 2-3 groups; Each timeline represents one research site;
**In Aim 3, FRAME: framework for reporting adaptations and modifications expanded[96]; IDI: In-depth Interview; FDG: Focus Discussion Groups

## Study Population

The study will enroll approximately 175-190 participants from clinics serving YPLWH across each of the four Tanzanian study sites (N=700-750), accounting for potential attrition.

## Inclusion and Exclusion Criteria:

- **Inclusion criteria**: youth between the ages of 10 and 24 years of age, who are attending the enrolling adolescent HIV clinic, are fully disclosed and aware of their HIV status, and are receiving ART for a minimum of 6 months; if $\geq$ 18 years, able to understand the project and provide written, informed consent; if < 18 years, a parent or guardian must provide written permission and participant must be able to assent; all adolescents must also commit to attending the 10 weekly SYV sessions and 2 individual sessions.
- **Exclusion criteria**: active psychosis, developmental delay, or cognitive disability that precludes active participation in consent process, intervention, and assessment interviews.

## Sample size calculation

We will individually randomize up to 700-750 participants divided across the four sites to receive the SYV intervention or standard of care across four sites in Tanzania. This sample size calculation is based on a plausible proportion of 80% viral suppression (VS) in standard of care and 90% VS in SYV intervention at the primary time point (6-months post-baseline ($T_2$)). In the original pilot trial, the VS rate at follow-up was 65% in SOC and 75% in SYV. Because the roll-out of dolutegravir and other factors may lead to increased baseline VS rates, here we assume a reference rate of 80% VS in SOC at 6-months post-baseline. We also assume a conservative ICC estimate of 0.04 (noting that the ICC for viral suppression was ~0.04 in the original pilot data), and assume 8 participants per group in the SYV intervention would have data available for analysis at $T_2$. With these assumptions, we have 90% power at a two-tailed significance (alpha) level of 0.05 to detect this 10-percentage point effect with 264 participants (33 groups of 8 participants each) in intervention and 311 participants in standard of care (575 participants). Assuming 10% attrition by the primary time point in both arms (noting the 91% retention rate in the original pilot trial), we increase the group size in intervention to 9 (297 participants in 33 groups) and the number of participants in standard of care to 346, which leads to a final total sample size of 643 across

both arms (Table 2, below). We used a power calculation specifically designed to account for the individually randomized group treatment trial design[77]. Moreover, we note that for this study design, statistical power requires unequal sample sizes (i.e., larger sample size in SOC) because of clustering in the intervention arm. In practice and if feasible, we will increase the number of participants in each group in the intervention arm to 10-11 (49 additional participants spread across the 33 groups), thus eliminating the imbalance between arms, so that there are 346 participants in intervention and 346 in control. We may also increase sample size later in the trial if dropout in earlier waves exceeds 10%.

**Table 2:** Power for expected effect size for primary outcome of Aim 1.

| | Original | Equal N per arm |
|---|---|---|
| Power | 90% | >90% |
| Alpha | 5% | 5% |
| Dropout Rate | 10% | 10% |
| Proportion VS in SYV Intervention | 90% | 90% |
| Proportion VS in Standard of Care | 80% | 80% |
| Intra-cluster correlation (ICC) | 0.04 | 0.04 |
| Number of participants per group | 9 | 10-11 |
| Number of participants in SYV | 297 | 346 |
| Number of participants in SOC | 346 | 346 |
| Total Number of participants* | **643** | **692** |

*Number recruited and randomized

### Sampling technique/Recruitment

**Pilot.** Collaborating researchers from Tanzania will identify and recruit 8 pilot study participants at each site. Pilot study participants will be recruited based on age categorization specified at each site as well as having HIV suppression at most recent standard of care HIV RNA test. After recruitment, data collection for the pilot proceeds in the same way as described below for the parallel-arm RCT, beginning with screening. Biologic specimens will **not** be obtained during the pilot, but the staff who will collect blood and hair samples during the RCT will be asked to pretend by meeting the participant and labeling a fake sample and taking this to the laboratory to ensure these logistics work smoothly.

**Parallel-arm RCT.**
*Recruitment.* Participants will be recruited from the HIV care and treatment centers that serve YPLWH ages 10-24 years of age, focusing on those 15-24, across the four participating sites in Tanzania. The study will be explained to the center staff and presented to YPLWH in the form of a health talk to the group and/or individually by a study team or staff member who already works in or is a former or current patient in the clinic (for example, a study supervisor

or young adult group leader). This is to avoid any unintended HIV disclosure of youth to study personnel who may not routinely be present in the clinic.

*Screening.* Youth interested in the study will be screened at a participating clinic to determine if they are eligible. During screening age, sex, contact information, medical record number (MRN), and eligibility criteria data are collected. Direct identifiers are collected during screening for purposes of identifying unique individuals and contacting eligible individuals in the future. Screening is conducted by collaborating researchers in Tanzania; no Duke personnel or resources are involved. If a youth screens eligible, they are invited to consent, or to assent with parental consent if younger than 18. A home visit can accompany the consent/assent process with the youth and their caregiver as desired by both parties (youth, caregiver, and study team). The study team will obtain caregiver consent either in person or if the caregiver is unable to accompany the youth to clinic and refuses a home visit the youth may take the consent form home for caregiver signature. In this case, the research team will call the caregiver to explain the study and answer any questions, the caregiver will sign once all questions have been answered and the youth may return the form to study staff. If the youth screens ineligible or declines assent/consent, then their screening data **excluding** direct identifiers is transcribed into REDCap. This is for the purpose of proper reporting of participant flow and reasons for ineligibility or decline. If an eligible youth assents/consents, then their screening data **including** direct identifiers is transcribed into REDCap. Youth who consent into the study are added to a waitlist. The waitlist is used to ensure a set of 20-22 youth of similar age and same sex is assembled and available prior to randomization. The waitlist will be maintained throughout the study enrollment period (years 2 - 4 of the study) and interested youth can add their name on a rolling basis. Being on the waitlist does not obligate a youth to participate.

*Baseline Data Collection*. Sixty days prior to the start of a new wave, participants on the waitlist within each site will be assembled into two separate sets of 20-22 according to: age, to ensure developmentally similar groups; gender to ensure a full set of males and a full set of females are available for randomization; and anticipated HIV RNA based on SOC data. Once 20-22 youth have been identified for a set, they are enrolled and the baseline data collection event will occur. A research assistant will administer questionnaires (Appendix 1) to participants in one-on-one structured interviews and capture responses using the REDCap Mobile application running on a tablet device. Biologic specimens (blood and hair samples) will also be collected by research assistants and a trained phlebotomist. After the interview and sample collection, the research assistant will abstract data from the participant's medical chart.

*Randomization.* Randomization will occur just after baseline data collection for participants in the set. This is to prevent attrition. Each set of 20-22 participants will be randomized in a 1:1 ratio to intervention and control as specified in the **Randomization** section below. Youth randomized to the SYV intervention will be notified by phone call or SMS message confirming time and date of the session. A home visit, if acceptable to youth and caregiver,

may also be offered to further explain the intervention or to notify the youth and family of their randomization status.

*Follow-up Data Collection.* Questionnaire data, biologic samples, and medical chart abstraction data are collected at 4-, 6-, 12-, and 18-months post baseline, in the same manner as they were collected at baseline.

Participants in the SOC will not meet in study groups, thus are more “at risk” for attrition. They will be contacted by the study team on a monthly basis to check in and ensure their documented contact information remains accurate. SOC may vary by site depending on clinic structure, referral systems, and group activities. These differences could potentially dilute the SYV intervention effect and introduce content spillover whereby participants randomized to the intervention discuss intervention content with participants randomized to SOC. As part of the study questionnaire, we will ask participants if they had friends who attended the SYV intervention and if they discussed content. Additionally, as part of the implementation science outcomes (Aim 3), the SOC group structure, adherence counseling, and any mental health referrals offered as part of SOC will be documented. On an individual level, participation in enhanced adherence counseling, mental health referrals, or additional group activities will be documented for both participants randomized to the SYV arm or to the SOC arm.

**Data Collection and Management**

Each site will have an internet technology and data management specialist available on site to supervise data collection and management. A data architect will work closely with all site teams and statisticians to coordinate the multifaceted data collection apparatus, including the multiple platforms (REDCap mobile, NVivo, DukeBox) used to collect and manage data. The data architect will oversee data collection workflow, implement data quality controls, report on data collection progress, align data encoding with the statistical analysis plan to reduce the data cleaning burden on statisticians, and maintain metadata such as data dictionaries for a future data repository deposit. Data transfer agreements and/or similar contracts will be signed across all sites with investigators as needed and appropriate to facilitate access to primary research data and samples during and after the study.

Further information on collection and management of specific data types is described below.

*Questionnaire data.* A research assistant will administer questionnaires to participants in one-on-one sessions at the various data collection timepoints (see Table 1 and Table 4). The research assistant will record participant responses using the REDCap Mobile application running on a tablet device. Completion of the questionnaire is expected to take approximately 60-90 minutes. Raw questionnaire data will be uploaded from tablets and stored in the project’s dedicated REDCap database, provisioned through the Duke Office of Clinical Research (DOCR). Questionnaire data will be exported out of REDCap to a dedicated project folder on DukeBox for data management processes including but not limited to: quality control, centralized processing, creation of the analytic dataset, and

reporting. Both the DOCR-maintained instance of REDCap and DukeBox are HIPAA-compliant. See *Appendix A* for full interview questionnaire.

*Medical chart abstraction*. A research assistant will abstract data from participant medical charts at each data collection timepoint. Data to be abstracted include direct identifiers (medical record number, date of birth, dates of medical tests) and health information related to HIV diagnosis, care, and treatment. See *Appendix A,* instrument *Chart,* for complete list of data abstracted.

*Biologic samples*. Biologic samples will also be collected at each data collection timepoint. A 5-10 mL blood sample will be collected for HIV RNA with an aliquot stored at the local laboratory (as noted in the consent form) for the possibility of future viral genotype testing and/or testing markers of inflammation. A small amount of hair (20-30 strands) will be cut with scissors from the occiput scalp near the follicle to provide an objective measure of adherence detecting ART concentration in the body. This measure provides an objective measure of adherence over a longer duration of time (weeks to months)[78]. The blood and hair collection methods for this study have been acceptable in our prior studies with YPLWH in Tanzania and are expected to be acceptable in this study[79,80].

*Economic data*. Data for cost-effectiveness will quantify direct and indirect costs using a patient cost survey as well as financial reports from the project. Direct costs include service delivery personnel, supplies, laboratory investigations, and patient medical costs. Indirect costs include training, management, demand generating activities, equipment, overhead (e.g. administration, indirect personnel, building, and general equipment), patient transportation, and loss of economic productivity. Data to estimate resource utilization and personnel time will be collected through a time and motion study and review of facility/SYV program financial reports on a monthly to quarterly basis. Volunteer time will be valued at a minimum domestic service wage rate for Tanzania. Financial reports to estimate hourly wages and percentage of time spent conducting trainings for SYV personnel we be used to derive a component of the intervention delivery cost. Participants will be asked about cost related to HIV care in a survey administered at each study timepoint (Table 1) to estimate any cost difference between the SYV and SOC groups. Direct and indirect cost data collected through these processes will be used to estimate the unit cost per beneficiary of the SYV intervention.

*Qualitative data.* Qualitative data collection for Aim 3 will be collected using Indepth Interviews (IDIs) to capture more individual experiences and Focus Discussion Groups (FDGs) to get the group perspective in order to answer the implementation science objectives related to acceptability, feasibility and sustainability among key stakeholders. Using the CFIR implementation science framework, stakeholders from key domains will be interviewed to better understand implementation of the SYV intervention across sites and how to best position the intervention for integration and sustainability if it is effective. Key stakeholder IDI and FDG will occur across the listed domains (see Table 1). Examples of key stakeholders or members of these domains include, but are not limited to: 1) outer setting: ministry of health or local NGOs or youth community advisory board; 2) inner setting:

head of adolescent HIV clinic at each site, the study PI, the study supervisor, social worker, others that work within the site adolescent HIV clinic; 3) key individuals: group leaders and SYV participants; 4) Process: will be monitored using fidelity, adapatations through the FRAME framework. IDIs and FDGs will be conducted by trained Tanzanian qualitative researchers. The IDI and FDG will use semistructured interview guides derived from domain maps tailored to the objectives of each stakeholder group. IDI and FDG will be audio-recorded with participant consent and conducted in Kiswahili for the key individuals (participants of SYV and group leaders) and the preferred language choice of English or Kiswahili for the inner and outer setting (head of Tanzania UNICEF, MOH, study PI, etc).

Data for Aims 1, 2 and 3, including qualitative and economic data, will be stored securely on Duke Box with key personnel on the study team from all sites able to access de-identified and password protected quantitative data and qualitative transcripts (see Data Management section below for more details).

**Randomization**

Randomization will occur by computer-generated permuted blocks of size 4 generated using SAS software. Randomization will be performed separately at each site, and stratified by sex and enrollment HIV RNA (<400 copies/mL or ≥400 copies/mL) to ensure balance on sex, proportion with virologic suppression across both arms. Those randomized to receive the SYV intervention will be assembled into 33 groups of 10-11 participants per group (Table 3). Those randomized to SOC will continue to receive routine care, which will be clearly defined by each site. The outcomes assessor and statisticians will be masked to participant study arm through the 6-month study visit (primary aim). Randomization will be performed at a central location (Duke) by the lead data architect, so individual sites will not have access to the randomization tables and thus will be blinded to randomization ahead of time to reduce the risk of selection bias.

**Table 3:** Number of participants per randomizaiton set, wave, and site**

| SITE | Wave 1 | | Wave 2* | | Wave 3* | | Wave 4* | |
|---|---|---|---|---|---|---|---|---|
| Ifakara (N=160-176) 8 sets | Set 1 (N=20-22) | SYV (N=10-11) | Set 3 (N=20-22) | SYV (N=10-11) | Set 5 (N=20-22) | SYV (N=10-11) | Set 7 (N=20-22) | SYV (N=10-11) |
| | | SOC (N=10-11) | | SOC (N=10-11) | | SOC (N=10-11) | | SOC (N=10-11) |
| | Set 2 (N=20-22) | SYV (N=10-11) | Set 4 (N=20-22) | SYV (N=10-11) | Set 6 (N=20-22) | SYV (N=10-11) | Set 8 (N=20-22) | SYV (N=10-11) |
| | | SOC (N=10-11) | | SOC (N=10-11) | | SOC (N=10-11) | | SOC (N=10-11) |
| KCMC/ Mawenzi (N=160-176) 8 sets | Set 1 (N=20-22) | SYV (N=10-11) | Set 3 (N=20-22) | SYV (N=10-11) | Set 5 (N=20-22) | SYV (N=10-11) | Set 7 (N=20-22) | SYV (N=10-11) |
| | | SOC (N=10-11) | | SOC (N=10-11) | | SOC (N=10-11) | | SOC (N=10-11) |
| | Set 2 (N=20-22) | SYV (N=10-11) | Set 4 (N=20-22) | SYV (N=10-11) | Set 6 (N=20-22) | SYV (N=10-11) | Set 8 (N=20-22) | SYV (N=10-11) |
| | | SOC (N=10-11) | | SOC (N=10-11) | | SOC (N=10-11) | | SOC (N=10-11) |
| Mbeya (N=160-176) 8 sets | Set 1 (N=20-22) | SYV (N=10-11) | Set 3 (N=20-22) | SYV (N=10-11) | Set 5 (N=20-22) | SYV (N=10-11) | Set 7 (N=20-22) | SYV (N=10-11) |
| | | SOC (N=10-11) | | SOC (N=10-11) | | SOC (N=10-11) | | SOC (N=10-11) |

| | | | | | | | | |
|---|---|---|---|---|---|---|---|---|
| | Set 2 (N=20-22) | SYV (N=10-11) | Set 4 (N=20-22) | SYV (N=10-11) | Set 6 (N=20-22) | SYV (N=10-11) | Set 8 (N=20-22) | SYV (N=10-11) |
| | | SOC (N=10-11) | | SOC (N=10-11) | | SOC (N=10-11) | | SOC (N=10-11) |
| Mwanza (N=160-176) 8 sets | Set 1 (N=20-22) | SYV (N=10-11) | Set 3 (N=20-22) | SYV (N=10-11) | Set 5 (N=20-22) | SYV (N=10-11) | Set 7 (N=20-22) | SYV (N=10-11) |
| | | SOC (N=10-11) | | SOC (N=10-11) | | SOC (N=10-11) | | SOC (N=10-11) |
| | Set 2 (N=20-22) | SYV (N=10-11) | Set 4 (N=20-22) | SYV (N=10-11) | Set 6 (N=20-22) | SYV (N=10-11) | Set 8 (N=20-22) | SYV (N=10-11) |
| | | SOC (N=10-11) | | SOC (N=10-11) | | SOC (N=10-11) | | SOC (N=10-11) |

*same numbers expected in each Wave, set, and randomization arm
**in practice, one site will have an extra set to be able to obtain the 33 sets required per the sample size calculation.

### Study Variables and Outcomes

Study variables are summarized in **Table 4** and listed comprehensively in *Appendix A*. The primary outcome is virologic suppression (measured as HIV RNA <400 copies/mL) at $T_2$. Secondary outcomes include HIV RNA at other timepoints; ART adherence based on concentration from hair samples and self-report in the intervention arm, and also **1)** mental health; **2)** coping; **3)** resilience; **4)** stigma; **5)** quality of life; **6)** gender based violence; **7)** disclosure; **8)** HIV knowledge; and **9)** high-risk behaviors (sex without a condom and substance use), which will be assessed at $T_0$, $T_1$, $T_2$, $T_3$, and $T_4$ (baseline, 4-, 6-, 12-, 18-month follow-up) (see **Table 1**). Exploratory outcomes include viral genotype testing (if HIV RNA ≥400 copies/mL) and biologic markers of inflammation.

### Laboratory Considerations

As we have done in the original SYV pilot study in Moshi, we plan to collect blood samples (10 mL) for HIV-1 RNA and resistance testing as well as a small amount of hair samples (20-30 strands of hair) which is cut near the root, but causes no pain to the participant. More hair falls out naturally in a day than that collected for study and in our past research has been well accepted by the youth.

Blood collected will be analyzed for HIV RNA at the local laboratory. Samples will be stored locally at -80$^0$ C until the study is complete. For the purposes of this study, samples are expected to be stored for approximately four years. Remaining samples at study completion will be retained at the discretion of the local research laboratory and any use must be within the informed consent agreement. Consent will be obtained for storage/biobanking and potential exploratory investigations either as part of SYV or otherwise for use by the local laboratory. If there is funding for HIV Genotype to evaluate HIV resistance mutations these studies will be performed in Tanzania if the technology is available. Hair samples are collected and placed in aluminum foil. The samples will be analyzed at the University of California San Francisco laboratory as the technology of drug concentration is not currently available in East Africa. Unless otherwise specified by local sites, excess samples received at UCSF will be destroyed. Appropriate material transfer agreements will be in place for any sample shipments.

**Table 4:** Study measures and outcomes based on the modified SAT framework[58,59]

| Domain | Construct | Tool | Administration* |
|---|---|---|---|
| Contextual Factors | Demographics | Participant: Age, sex, tribe, education, employment, perceived health status; children, pregnancy, marriage<br>Related to the primary caregiver (as answered by youth): relation to the primary caregiver, primary caregiver's age, sex, employment, education, marital status, perceived physical and mental health status | $T_0$ |
| | Environment | Death of parent(s); if so, due to what cause; home environment (electricity, plumbing, number of rooms); trauma in the home environment is captured in the UCLA Post Traumatic Stress Screen | $T_0$ |
| | Disclosure | Purposefully told versus found out on own; How HIV was acquired (perinatal, sexual, other, unknown) | $T_0$ |
| | Change in Baseline factors | Primary caregiver, home environment, school, employment, perceived health status, children, pregnancy, marriage | $T_1$, $T_2$, $T_3$, $T_4$ |
| Self and social regulation | Mental Health | Patient Health Questionnaire (PHQ-9)[81,82] | $T_0$, $T_1$, $T_2$, $T_3$, $T_4$ |
| | | Strengths and Difficulties Questionnaire (SDQ)[83] | $T_0$, $T_1$, $T_2$, $T_3$, $T_4$ |
| | | Adverse Childhood Events-International Questionnaire and UCLA Trauma Reaction Index survey[84,85] | $T_0$, $T_1$, $T_2$, $T_3$, $T_4$ |
| | | Generalized Anxiety Disorder-7 Questions (GAD-7)[86] | $T_0$, $T_1$, $T_2$, $T_3$, $T_4$ |
| | Coping | The brief COPE[87] | $T_0$, $T_1$, $T_2$, $T_3$, $T_4$ |
| | Resilience | People Living with HIV Resilience Scale[88] | $T_0$, $T_1$, $T_2$, $T_3$, $T_4$ |
| | Stigma | 10 question stigma scale[89] | $T_0$, $T_1$, $T_2$, $T_3$, $T_4$ |
| | Quality of Life | WHOQOL-HIV-BREF[90] | $T_0$, $T_1$, $T_2$, $T_3$, $T_4$ |
| | Gender based violence | WHO Victim/Perpetrator[91] | $T_0$, $T_1$, $T_2$, $T_3$, $T_4$ |
| | Disclosure | Purposefully disclosed to someone new (if yes, who and why) | $T_0$, $T_1$, $T_2$, $T_3$, $T_4$ |
| | HIV knowledge | HIV knowledge[92] | $T_0$, $T_1$, $T_2$, $T_3$, $T_4$ |
| Behavioral Health Outcomes | **HIV RNA[x]** | **10mL blood (5mL held for exploratory outcomes with consent)** | $T_0$, $T_1$, $\mathbf{T_2}$, $T_3$, $T_4$ |
| | Adherence | Self-report, 3 Questions[93] | $T_0$, $T_1$, $T_2$, $T_3$, $T_4$ |
| | | ART levels in hair[94] | $T_0$, $T_2$ |
| | Behavior | Sexual activity and condom use | $T_0$, $T_1$, $T_2$, $T_3$, $T_4$ |
| | | Substance Use | $T_0$, $T_1$, $T_2$, $T_3$, $T_4$ |

## Statistical Methods

**Quantiative data Analysis**: Aim 1: In order to estimate risk differences and risk ratios comparing virologic suppression in SYV vs. SOC, we will use modified Poisson generalized estimating equation (GEE) models to examine the effect of intervention on virologic suppression at each follow-up time point. The GEE models will take into account clustering using an exchangeable working correlation in the SYV intervention arm, and will use Kauermann-Carroll corrected standard errors to take into account potential small-sample bias in the standard errors[95]. For continuous secondary outcomes, we will use linear mixed effects models. The mixed effects models will include a random intercept for group, where each individual in the SOC arm is considered a group of size 1, as is appropriate for IRGTs with clustering in one arm[96]. For continuous outcomes, we will use constrained longitudinal models which assumes that intervention arm means are equal at baseline, which increases power and is appropriate in a randomized design[97]. To take into account that type I error is generally inflated when standard denominator degrees of freedom are used for hypothesis testing when the number of clusters is <40, we will use the between-within method to estimate the denominator degrees of freedom for the hypothesis tests[98]. All analyses will be based on the intention-to-treat principle whereby all participants will be included in the analysis irrespective of their level of involvement in the intervention. Missing data will be

taken into account by first examining baseline variables determined to be differential by dropout. These variables will then be included in the statistical models as a sensitivity analysis, under the assumption that the data are missing at random conditional on the baseline variables.

Additionally, we will measure compliance (i.e., number of sessions attended in the intervention arm) and spillover (i.e., amount of information from group sessions that control participants learn), and will use causal inference methods to examine the complier average causal effect (CACE), as well as the effect of spillover.

Aim 2: To evaluate mediation and moderation, we will first examine the treatment effect on four continuous mediators (mental health first limited to PHQ-9) coping, resilience, and internal stigma, based on the intention-to-treat principle whereby all participants are included in the analysis irrespective of their level of involvement in the intervention. To ensure the time aspect of causal mediation, we will examine the effect of intervention on the mediators at 4-months ($T_1$), and then the effect of the mediators on ART adherence and HIV RNA at 6 months ($T_2$). We will model the mediators jointly as correlated parallel mediators and estimate their combined and unique indirect effects using the structural equation modeling (SEM) framework. We will estimate combined and unique indirect effects of our correlated mediators within the SEM framework[99], with HIV RNA dichotomized (virologic suppression <400 copies/mL) as the outcome. Clustering of mediators and outcomes in the SYV arm will be accounted for, as well as potential confounding of the mediator-outcome relationships in all analyses. Effect modification will be analyzed by including each moderator separately (age, sex, site, severity of mental health symptoms at baseline) in the Aim 1 statistical model with virologic suppression as the outcome, and including the interaction between intervention arm and the moderator. Exploratory analyses of participants randomized to SYV will compare outcomes according to degree of attendance to generate hypotheses around potential dose-response relationships.

Aim 3: Implementation science related to cost effectiveness and sustainability: To evaluate implementation outcomes we will analyze data using mixed methods of quantitative and qualitative data. Cost effectiveness we will use an Incremental Cost-Effectiveness Ratio (ICER), defined as the difference in costs between SYV and SOC divided by the difference in effectiveness. In accordance with the recommendations of the second panel on cost-effectiveness in health and medicine, we will develop a reference case that takes both the societal and health systems perspective. We will estimate the unit cost per young person living with HIV (YPLWH) randomized to receive the Sauti ya Vijana (SYV) intervention, and the unit cost per YPLWH randomized to receive standard of care (SOC) using micro-costing methods.

**Qualitative analysis (Aim 3):**
Qualitative data analysis will be informed by a grounded thematic analytical approach to understand barriers and facilitators of implementation and key outcomes including acceptability, feasibility, and sustainability. Interviewers who conduct the IDI and FDG will take field notes; transcribe Kiswahili audio, and translate Kiswahili to English transcripts.

This process will be verified by a second fluent Swahili and English speaker to ensure meaning is consistent and that no direct identifiers are be retained. IDI's (approximately N=70 over the course of the study), FDGs (N=15-25), and weekly session notes combined per intervention site and wave (N=33-35). Qualitative data will be analyzed and interpreted by a team including expert social scientists from the US and Tanzania with desire to train social scientists at each site interested in helping collect, code, and write up these data. Qualitative data will be coded using NVivo software. Nvivo enables researchers to explore how theoretical concepts fit by developing and modifying a hierarchical coding index. Thematic analysis will be conducted via an iterative process of data collection and analysis that utilizes four interrelated steps: reading; coding; data display; and data reduction[100].The team will use a codebook of theoretically driven structural codes based on the CFIR and implementation outcomes framework. A second round of content coding will be conducted to identify additional themes, ideas, or concepts. A minimum of 40% of transcripts and session notes will be coded by two team members, interrater reliability assessed, and discussions held to resolve coding discrepancies.

<u>Quantitative and Qualitative data will also be integrated using data triangulation techniques towards aim 3 regarding implementation science</u>. Data triangulation is often used for analysis when multiple sources, theories, and researchers are involved, thus enhancing the research rigor and findings' credibility[101,102]. For example, 'acceptability' will be measured via multiple data sources (IDIs, FDGs, supervision notes, attendance, retention, etc.) and these data can be analyzed and interpreted using a triangulation protocol that results in a meta-theme on acceptability rather than a listing of results per data source. Matrices will help organize the data by source or theoretical framework for each barrier, facilitator, and outcome.

## ETHICAL CONSIDERATIONS

### Institutional Ethical Clearance

This protocol, the informed consent forms, and any subsequent modifications will be reviewed and approved by the Institutional Review Board of Duke University Medical Center, Baylor Center of Excellence, the Ethics Committee of Kilimanjaro Christian Medical Center, as well as ethics boards at Ifakara health institute, Mwanza, and Mbeya and together at the Tanzanian National Institute of Medical Research with respect to scientific content and compliance with all applicable research and human subjects regulations. The investigators will make safety and progress reports to these IRBs as required by each IRB.

**Risks to Subjects:** Approval from each institutionwill be obtained before any subject recruitment or enrollment. As this study is a behavioral intervention, the risk is very low to the patient and is not beyond that expected from routine clinical care.

**Patient Benefits and Follow-up of Abnormal Findings:** The benefits of this study are that the mental health, coping skills, and ART adherence of participants are likely to improve. If the study team has reason to believe a participant is suicidal, the participant will be promptly referred to psychiatric care within the local standard of care. Participants will receive travel reimbursement for each study visit and a food snack as part of the weekly intervention.

## Confidentiality/De-identifying data:

The study will collect PHI, including the direct identifiers: name, telephone number, address (at level of village/ward), date of birth, medical record number (MRN), dates of medical tests and test results, and voice prints (from focus group discussion and in-depth interview audio recordings). Some PHI is collected on paper records and accessible only by in-country team members. Some PHI is collected using the REDCap Mobile application as part of research data collection. Specifics are described below.

*Electronic Data Records*. The study will collect PHI with Duke REDCap and the REDCap Mobile app, including direct identifiers: address (at level of village/ward), date of birth, medical record number (MRN), dates of medical tests and test results. A unique study ID will be assigned to the participant during their first data collection event. Study personnel directly involved with data collection will have access to identifiable information stored in REDCap. The Duke data manager will create and maintain analytic data files by processing data stored in Duke REDCap to remove direct identifiers and remove or transform potentially indirectly identifying values in the data. Such analytic data files will be stored on DukeBox, where study team personnel not directly involved in data collection may access them. We will conduct analysis using these de-identified analytic data files.
Audio files will be collected by the in-country study team and stored on DukeBox.
Technologies storing study participant PHI (Duke REDCap, DukeBox) are compliant with HIPAA and other local, institutional, and national policies, regulations, and laws governing sensitive data.

*Physical records*. The study will collect PHI on paper records, including direct identifiers: name, telephone number, and medical record number. Physical records, including but not limited to consent forms and paper-administered questionnaires, will be kept in a secured area behind locked doors. Laboratory specimens, evaluation forms, reports, and other records will utilize study codes instead of direct identifiers whenever possible to maintain subject confidentiality. Laboratory specimens will be stored securely as described above.

*Retention*. Data files will be retained for 6 years following the completion of the study or when participant turns 21, whichever is later. Any research information in the subject's medical record will be kept indefinitely.

*Sharing.* De-identified data will be shared with other researchers in compliance with funder (NIMH) data sharing requirements. De-identified data will be submitted bi-annually while data collection is ongoing to the National Institute of Mental Health (NIMH) Data Archive, with the first submission six months after the beginning of quantitative data collection. De-identified data may also be shared through other mechanisms, including but not limited to deposit in additional data repositories or through contractual agreements such as Data Use Agreements (DUAs). Prior to sharing, the Duke data manager will procure external review services verifying satisfactory de-identification from a qualified entity such as Duke's Center for Data and Visualization Services (CDVS).

*Exceptions*. Information about participants who are at the risk of causing self-harm or harm to others will be shared based on the study protocol and in accordance with Tanzanian guidelines. Identifying information may be shared as required by a legal or regulatory body overseeing the study, including but not limited to such bodies as a data safety monitoring board (DSMB).

## Consent:

All participants will be required to give informed consent. For those under 18, patient assent will also be obtained. Caregivers may accompany their youth for the informed consent

process or this may be done by telephone call with the study coordinator to answer any questions. Caregiver travel will be reimbursed. A home visit to further explain the study is also possible if agreed upon by the participant and their family. Full disclosure will be given to each subject in both written and oral form (with a witness as necessary); the purposes of the study will be explained to each participant. All risks, as outlined above, will be clearly expressed to each participant. Participation will be voluntary and each participant will be able to drop out at any time for any reason.

**Transcription and translation**
All study documents will be translated to Kiswahili and back translated to English to ensure the meaning of the information remains consistent across languages. These documents include 1) consent and assent forms, 2) study questionnaires encompassing the mental health measures, adherence questions, stigma, behavior, and HIV knowledge questions have been adapted to the local context in prior focus groups, and 3) the IDI and FGD semi-structured guides. Translation to Swahili occurred by a professional team (services rendered by Zacharia Massawe, Michelle Mshiu, and Leonia Rugulabamu). These documents can be found in the consent form and questionnaire appendices respectively.

## LIMITATIONS OF THE STUDY

Obtaining consent/permission from parents and guardians for youth <18 years may present a challenge, though we will reimburse travel for each parent and participant and provide a meal coupon to offset the cost of coming to clinic and potentially missing lunch. Participants randomized to SYV will return nearly weekly for 3 months, and anyone who enrolls as a minor and reaches 18 years will be re-consented as an adult. Life events may affect the participants' answers between survey administration and may affect test-retest reliability.

We may find that during the process of administering the questionnaire, we identify an individual with previously unrecognized mental health problems requiring referral to a mental health professional. If this occurs, the study staff will promptly provide that information to the participant's medical provider and pursue appropriate mental health care referral within the local standard of care.

## INVESTIGATOR ROLES

### <u>Kilimanjaro</u>

**Blandina Mmbaga, Principal Investigator at KCMC.** Director, Kilimanjaro Clinical Research Institute (KCRI) at KCMC. Professor Mmbaga will serve as Site Principal Investigator. She will help oversee the proposed research in the Kilimanjaro region, KCMC and Mawenzi sites. She will also be responsible for laboratory activities (HIV RNA) performed at the KCRI Biotechnology Laboratory at KCMC.

**Dorothy Dow, PI of overall study.** Dr. Dow will help cooridinate the KCMC site and other three Tanzanian sites. She will help supervise, trouble shoot logistic challenges, and help run the overall trial.

**Judith Boshe, Mental Health specialist (Supervisor).** Dr. Boshe is the psychiatrist at KCMC. She will lead the initial SYV training and help supervise group leaders. She will also participate in weekly research meetings to offer any additional comment to the session discussions and/or participant questions that arose. In addition, she will see participant referrals should a participant be found to have significant mental health challenges such as suicidal ideation.

**Aisa Shayo, Pediatrician (Co-Investigator).** Dr. Shayo will help refer YPLWH, supervise group leaders, and ensure study results make it into participant files.

**<u>Ifakara</u>**
**Getrud Mollel, MD.** Dr. Mollelwill serve as Site Principal Investigator. She will help oversee the proposed research in the Ifakara, Morogoro region. She will also be responsible for overseeing laboratory activities (HIV RNA) performed at the local laboratory.

**<u>Mwanza</u>**
**Judith Gwimile, MMED.** Dr. Gwmile will serve as Site Principal Investigator. She will help oversee the proposed research in the Mwanza region. She will also be responsible for laboratory activities (HIV RNA) performed at the laboratory.

**Bernard Desderius, MD.** Dr. Desderius will be a co-investigator offering support from the Bugando HIV clinic for recruitment of participants and young adult group leaders.

**<u>Mbeya</u>**
**Jason Bacha, MD.** Dr. Bacha will serve as Site Principal Investigator. He will help oversee the proposed research in the Mbeya region. He will also be responsible for laboratory activities (HIV RNA) performed at the local laboratory.

## DISSEMINATION OF THE RESULTS

Findings from this study will be presented to local and national stakeholders including clinic staff, YPLWH as part of the recruitment clinics, the community advisory boards and youth community advisory boards and national policy makers. Findings may also be presented at academic conferences in local and international settings and submitted for publication to scholarly journals where invited and/or accepted. Results will also shared at the community advisory board meetings and local social behavioral sciences conference. Budget for both domestic and international travel is available for site PIs and investigators to present results from their site in addition to the study team presentation of aggregated data.

## DETAILED BUDGET and BUDGET JUSTIFICATION
## SUMMARY TABLE OF BUDGET per site*

| | Year 1 | Year 2 | Year 3 | Year 4 | Year 5 | Total |
|---|---|---|---|---|---|---|
| Personnel costs | 24,000 | 24,000 | 24,000 | 24,000 | 24,000 | 120,000 |
| Consultant | 2,200 | 2,200 | 2,200 | 2,200 | 2,200 | 11,000 |

| | | | | | | |
|---|---|---|---|---|---|---|
| Site Expenses | 3,600 | 3,600 | 3,600 | 3,600 | 3,600 | 18,000 |
| Intervention | 2,970 | 2,970 | 2,970 | 2,970 | 2,970 | 14,850 |
| Samples | 12,750 | 12,750 | 12,750 | 12,750 | 12,750 | 63,750 |
| Travel | 1,000 | 1,000 | 1,000 | 1,000 | 1,000 | 5,000 |
| **Direct** | **46,520** | **46,520** | **46,520** | **46,520** | **46,520** | **232,600** |
| Indirect | 3,722 | 3,722 | 3,722 | 3,722 | 3,722 | 18,610 |
| **Total** | **50,242** | **50,242** | **50,242** | **50,242** | **50,242** | **251,210** |

*Note the KCMC budget is slightly higher for covering the cost of 1) training young adult group leaders for a two-week intensive meeting in Moshi; 2) the dissemination of results meeting; 3) hiring of an overall study coordinator; 4) retention of expert SYV group leaders to help supervise group leaders at all sites. The Ifakara budget includes slight increased funding for transportation of participants build into the intervention budget.

## Budget Justifications:

### Personel costs

**Site Principal Investigator**. The site PI will help oversee the proposed research in the their designated region. The site PI is responsible for laboratory activities (HIV RNA) performed at the local Laboratory. Support is requested for 1.2 calendar months (10%) effort.

**Local site supervisor (TBN).** Thesite supervisor will help oversee group leader training, practice sessions and supervise weekly research meetings. The supervisor will review and file fidelity checklists to session content, group leader observer notes, supervisor notes that comment on the ability of group leaders to effectively teach session content, and notes on the overall session discussion. The site supervisor may also participate in recruitment and consent procedures. Funds are requested to provide support for 4.8 calendar months (40%) effort.

**Group leaders (N=6).** The group leaders will be critical to the success of the intervention. They will be responsible for knowing the intervention manual content and their ability to teach the information effectively to the SYV participants. They will be responsible to keep information strictly confidential within the study team, to know their limitations in training and able to seek help from the supervisor team for difficult cases. They will attend an intensive training, practice the session content until mastery, then deliver the content over the course of the study. They will take session notes and mark the intervention fidelity check list. They will present challenges to the supervision team for guidance. Support is requested for 6 calendar months (50%) effort.

**Research Assistant (TBN).** The research assistant will assist in screening potential participants, ensuring ACASI questionnaire system is working properly to capture youth questionnaires, conduct the enrollment and subsequent follow up visits. The research assistant will also coordinate with the phlebotomist for blood and hair collection. Should the ACASI system fail to operate, the RA will read aloud the questionnaire (as would occur in ACASI) and the participant will complete the paper version marking their own answers in confidence. The RA will then key in responses to the database. The research assistant will

manage the transportation reimbursement of participant study visits. The research assistant is blinded to treatment arm. Position is 6.0 calendar months (50%) effort.

**Data Manager.** The data manager will be responsible for ensuring tablets and ACASI are working well, that data uploads to the REDCap database correctly and that data quality Is maintained. The data manager will also be responsible for ensuring supervisor notes and fidelity checklists are scanned and housed on Duke Box. All wave notes will be compiled and loaded to nVivo for qualitative data coding. The data manager will provide study supervisor with monthly data reports, help ensure study visits are on schedule, and maintain the information technology systems that support data collection and management (e.g., servers, computers, software). Funds are requested to provide support for 4.8 calendar months (40%) effort.

**Administrative Assistant.** The administrative assistant will help with IRB submissions, ensure good clinical practice certificates are up to date for study staff, assist with finance administration, and assist with human resources and keeping study contracts in good order. Funds are requested to provide support for 0.6 calendar months (5%) effort.

**Consultant**
**Phlebotomist.** Will support the research team and work closely with the research assistant in scheduling study visits. The phlebotomist will collection blood (by performing venipunctures) and hair (crown of head with scissors) of participants during study visits. Funds are requested on a per participant basis and budgeted for 150 participants X 5 study visits each, hourly rate is $2.5 for 400 hours.

**Site expenses. $3,600 per year**
Funds at $1,200 are requested for study materials (as needed) printer, computer/tablet x2; $1,200 are requested to subsidize session delivery space; and $1,200 is requested for Office supplies such as paper, toner, pens, white board/teaching pad/easel.

**Intervention expenses. $2,970 per year**
Funds are requested for 75 participants randomized to intervention arm (13 intervention sessions: 10 group; 2 individual; 1 booster) and 75 participants randomized to control arm X 5 study visits. $3,900 is requested for participant travel reimbursement for intervention, $3,000 is requested for participant travel reimbursement for study visits, $300 is requested for Counselor travel reimbursement (for home visits as needed), $2,080 is requested for drinks and snacks as part of the intervention and $2,250 as a meal voucher for study visits. $300 is requested for final and booster session (food, drinks and certificates), $520 is requested for Phone credit vouchers for counselors/supervisor/research assistant (calling clients for reminders) and $2,500 is requested for youth CAB meeting expenses. Total $14,850 in 5 years.

**Laboratory Expenses. $12,750 per year**

Funds at $63,750 is requested for HIV RN—-performed on site ($85 per sample including supplies, testing and sample storage) for 150 participants at 5 time points over the 5-year study period.

**Travel Expenses. $3,000 per year**
Funds at $1,000 is requested for travel of the Principal Investigator/site supervisor for local research dissemination and updates with MOH; $2,000 is requested for study coordinator travel cost and per diem when visiting different sites

*Budget unique to the Kilimanjaro site
**Study Coordinator (TBN).** This person will oversee all four site activities and support the local investigators in logistical support, ensure data is captured, study visits are on target, research meetings occur, coordinate teleconference of multi-site supervisor meetings (monthly) and all site youth community advisor board meetings (every 3 months). The coordinator will support quality control of data in concert with the data manager at each site and help ensure rigor and reproducibility. This person will also monitor ethics approvals and Good Clinical Practice compliance of all research staff. This position requires travel to each site once per wave to ensure the study roll out is on track and running smoothly. Support is requested for 12 calendar months (100%) effort.

**Expert Group Leaders (Unique to Moshi site).** Expert group leaders have four years of experience running the SYV original pilot RCT. The will attend and help supervisor group leader training, deliver group sessions, write up session notes and track fidelity to the intervention on fidelity checklists. The position is consulting per hour during site supervision meetings and for training.

**Launch Training-Moshi, Tanzania (30 people traveling, 15 local for 2 weeks). $31,050–YEAR 1 ONLY**
Funds at $2,250 is requested for venue cost to launch the training, $7,500 is requested as transport expense for Principal Investigators, Supervisors, Group Leaders and Research Assistants from Mbeya, Mwanza, and Ifakara to Moshi, Kilimanjaro for the launch Training. $10,800 is requested for Participant accommodation cost. $3,500 is requested for participant lunch cost. $4,000 is requested for Dinner. $2,000 is requested for refreshments during training and $1,000 is requested for Training materials such as flip charts, marker pens, budges, notebooks, pens etc.

**Research Dissemination Meeting (30 people traveling, 15 local for 3 days). $21,735–YEAR 5 ONLY**
Funds at $1,200 is requested for venue cost, $7,560 is requested as transport expense for Principal Investigators, Supervisors, Group Leaders, Research Assistants, Data Manager and Study Coordinator from Mbeya, Kilimanjaro, Iringa, and Ifakara to Mwanza for the meeting. $4,950 is requested for Participant accommodation cost. $2,650 is requested for Participant lunch cost. $3,200 is requested for Dinner. $1,175 is requested for refreshments during meeting and $1,000 is requested for meeting materials such as flip charts, marker pens, budges, notebooks, pens etc.

**Indirect costs:**
All sites will receive 8% of direct costs for their budgets.

## REFERENCES

1. United Nations Population Fund (UNFPA). The Power of 1.8 Billion: Adolescents, Youth, and the Transformation of the Future. State of the World's Population. (2014). New York, New York. Retrieved at: https://www.unfpa.org . Last Accessed: 2 September 2019.
2. Gore FM, Bloem PJ, Patton GC, et al. Global burden of disease in young people aged 10-24 years: a systematic analysis. *Lancet.* 2011;377(9783):2093-2102.
3. The Joint United Nations Programme on HIV/AIDS (UNAIDS). The Youth Bulge and HIV. Available at www.unaids.org/en/resources/documents/2018/the-youth-bulge-and-hiv. Last accessed 28 November 2019.
4. UNAIDS. Youth and HIV Mainstreaming a three-lens approach to youth participation. Accessed on 17 August 2018. Accessed at http://www.unaids.org/en/resources/documents/2018/youth-and-hiv. 2018.
5. White AM. Understanding adolescent brain development and its implications for the clinician. *Adolesc Med State Art Rev.* 2009;20(1):73-90, viii-ix.
6. Patton GC, Sawyer SM, Santelli JS, et al. Our future: a Lancet commission on adolescent health and wellbeing. *Lancet.* 2016;387(10036):2423-2478.
7. Lowenthal ED, Bakeera-Kitaka S, Marukutira T, Chapman J, Goldrath K, Ferrand RA. Perinatally acquired HIV infection in adolescents from sub-Saharan Africa: a review of emerging challenges. *Lancet Infect Dis.* 2014;14(7):627-639.
8. Nachega JB, Hislop M, Nguyen H, et al. Antiretroviral therapy adherence, virologic and immunologic outcomes in adolescents compared with adults in southern Africa. *J Acquir Immune Defic Syndr.* 2009;51(1):65-71.
9. Bernays S, Jarrett P, Kranzer K, Ferrand RA. Children growing up with HIV infection: the responsibility of success. *Lancet.* 2014;383(9925):1355-1357.
10. Dow DE, Shayo AM, Cunningham CK, Reddy EA. Durability of antiretroviral therapy and predictors of virologic failure among perinatally HIV-infected children in Tanzania: a four-year follow-up. *BMC Infect Dis.* 2014;14:567.
11. Ferrand RA, Munaiwa L, Matsekete J, et al. Undiagnosed HIV infection among adolescents seeking primary health care in Zimbabwe. *Clin Infect Dis.* 2010;51(7):844-851.
12. Kariminia A, Law M, Davies MA, et al. Mortality and losses to follow-up among adolescents living with HIV in the IeDEA global cohort collaboration. *J Int AIDS Soc.* 2018;21(12):e25215.
13. Memiah P, Shumba C, Etienne-Mesubi M, et al. The effect of depressive symptoms and CD4 count on adherence to highly active antiretroviral therapy in sub-Saharan Africa. *Journal of the International Association of Providers of AIDS Care.* 2014;13(4):346-352.
14. Yang E, Mphele S, Moshashane N, et al. Distinctive barriers to antiretroviral therapy adherence among non-adherent adolescents living with HIV in Botswana. *AIDS Care.* 2018;30(2):224-231.
15. Bucek A, Leu CS, Benson S, et al. Psychiatric Disorders, Antiretroviral Medication Adherence and Viremia in a Cohort of Perinatally HIV-Infected Adolescents and Young Adults. *Pediatr Infect Dis J.* 2018;37(7):673-677.
16. Smith Fawzi MC, Ng L, Kanyanganzi F, et al. Mental Health and Antiretroviral Adherence Among Youth Living With HIV in Rwanda. *Pediatrics.* 2016;138(4).

17. Okawa S, Mwanza Kabaghe S, Mwiya M, et al. Psychological well-being and adherence to antiretroviral therapy among adolescents living with HIV in Zambia. *AIDS Care.* 2018;30(5):634-642.
18. Mutumba M, Musiime V, Lepkwoski JM, et al. Examining the relationship between psychological distress and adherence to anti-retroviral therapy among Ugandan adolescents living with HIV. *AIDS Care.* 2016;28(7):807-815.
19. Patel V, Flisher AJ, Hetrick S, McGorry P. Mental health of young people: a global public-health challenge. *Lancet.* 2007;369(9569):1302-1313.
20. Sankoh O, Sevalie S, Weston M. Mental health in Africa. *The Lancet Global health.* 2018;6(9):e954-e955.
21. UNAIDS. FAST-TRACK COMMITMENTS TO END AIDS BY 2030. Geneva, 2016. Available at: https://www.unaids.org/sites/default/files/media_asset/fast-track-commitments_en.pdf. Last accessed 4 December 2019.
22. Cohen MS, Chen YQ, McCauley M, et al. Prevention of HIV-1 infection with early antiretroviral therapy. *N Engl J Med.* 2011;365(6):493-505.
23. The Lancet H. U=U taking off in 2017. *The lancet HIV.* 2017;4(11):e475.
24. PHIA Project (Population-based HIV impact assessment). Last accessed 27 April 2020 on Data, Data Visualization, All countries, Viral load suppression, 15-24 years. Accessed at: https://phia-data.icap.columbia.edu/visualization
25. Brown LK, Kennard BD, Emslie GJ, et al. Effective Treatment of Depressive Disorders in Medical Clinics for Adolescents and Young Adults Living With HIV: A Controlled Trial. *J Acquir Immune Defic Syndr.* 2016;71(1):38-46.
26. Safren SA, Bedoya CA, O'Cleirigh C, et al. Cognitive behavioural therapy for adherence and depression in patients with HIV: a three-arm randomised controlled trial. *Lancet HIV.* 2016;3(11):e529-e538.
27. Andersen LS, Magidson JF, O'Cleirigh C, et al. A pilot study of a nurse-delivered cognitive behavioral therapy intervention (Ziphamandla) for adherence and depression in HIV in South Africa. *J Health Psychol.* 2018;23(6):776-787.
28. Bere T, Nyamayaro P, Magidson JF, et al. Cultural adaptation of a cognitive-behavioural intervention to improve adherence to antiretroviral therapy among people living with HIV/AIDS in Zimbabwe: Nzira Itsva. *J Health Psychol.* 2017;22(10):1265-1276.
29. Petersen I, Hanass Hancock J, Bhana A, Govender K. A group-based counselling intervention for depression comorbid with HIV/AIDS using a task shifting approach in South Africa: a randomized controlled pilot study. *J Affect Disord.* 2014;158:78-84.
30. Vreeman RC, McCoy BM, Lee S. Mental health challenges among adolescents living with HIV. *J Int AIDS Soc.* 2017;20(Suppl 3):21497.
31. Mellins CA, Malee KM. Understanding the mental health of youth living with perinatal HIV infection: lessons learned and current challenges. *J Int AIDS Soc.* 2013;16:18593.
32. McKay M, Block M, Mellins C, et al. Adapting a Family-Based HIV Prevention Program for HIV-Infected Preadolescents and Their Families: Youth, Families and Health Care Providers Coming Together to Address Complex Needs. *Soc Work Ment Health.* 2007;5(3/4):355-378.
33. Bhana A, Mellins CA, Petersen I, et al. The VUKA family program: piloting a family-based psychosocial intervention to promote health and mental health among HIV infected early adolescents in South Africa. *AIDS Care.* 2014;26(1):1-11.
34. Nestadt DF, Saisaengjan C, McKay MM, et al. CHAMP+ Thailand: Pilot Randomized Control Trial of a Family-Based Psychosocial Intervention for Perinatally HIV-Infected Early Adolescents. *AIDS Patient Care STDS.* 2019;33(5):227-236.
35. World health Organization (WHO). HIV and Youth. Last accessed August 14, 2020. Available at https://www.who.int/maternal_child_adolescent/topics/adolescence/hiv/en/.

36. Willis N, Milanzi A, Mawodzeke M, et al. Effectiveness of community adolescent treatment supporters (CATS) interventions in improving linkage and retention in care, adherence to ART and psychosocial well-being: a randomised trial among adolescents living with HIV in rural Zimbabwe. *BMC Public Health.* 2019;19(1):117.
37. Mavhu W, Willis N, Mufuka J, et al. Effect of a differentiated service delivery model on virological failure in adolescents with HIV in Zimbabwe (Zvandiri): a cluster-randomised controlled trial. *Lancet Glob Health.* 2020;8(2):e264-e275.
38. Fabri M, Ingabire C, Cohen M, Donenberg G, Nsanzimana S. The mental health of HIV-positive adolescents. *The lancet Psychiatry.* 2015;2(8):e21.
39. ClinicalTrials.gov [Internet]. Bethesda (MD): National Library of Medicine (US). Identifier NCT04024488 , Group-Based Intervention to Improve Mental Health and Adherence Among Youth Living With HIV in Low Resource Settings. Available at https://clinicaltrials.gov/ct2/show/NCT04024488.
40. Sawyer SM, Afifi RA, Bearinger LH, et al. Adolescence: a foundation for future health. *Lancet.* 2012;379(9826):1630-1640.
41. Caserta TA, Pirttila-Backman AM, Punamaki RL. Stigma, marginalization and psychosocial well-being of orphans in Rwanda: exploring the mediation role of social support. *AIDS Care.* 2016;28(6):736-744.
42. Kinyanda E, Hoskins S, Nakku J, Nawaz S, Patel V. Prevalence and risk factors of major depressive disorder in HIV/AIDS as seen in semi-urban Entebbe district, Uganda. *BMC Psychiatry.* 2011;11:205.
43. Lowenthal ED, Marukutira TC, Chapman J, et al. Psychosocial assessments for HIV+ African adolescents: establishing construct validity and exploring under-appreciated correlates of adherence. *PLoS One.* 2014;9(10):e109302.
44. Cluver L, Sherr L, Grimwood A, et al. Assembling an effective paediatric HIV treatment and prevention toolkit. *Lancet Glob Health.* 2014;2(7):e395-396.
45. Powell BJ, Patel SV, Haley AD, et al. Determinants of Implementing Evidence-Based Trauma-Focused Interventions for Children and Youth: A Systematic Review. *Adm Policy Ment Health.* 2019.
46. Murray LK, Skavenski S, Michalopoulos LM, et al. Counselor and client perspectives of Trauma-focused Cognitive Behavioral Therapy for children in Zambia: a qualitative study. *J Clin Child Adolesc Psychol.* 2014;43(6):902-914.
47. Woods-Jaeger BA, Kava CM, Akiba CF, Lucid L, Dorsey S. The art and skill of delivering culturally responsive trauma-focused cognitive behavioral therapy in Tanzania and Kenya. *Psychol Trauma.* 2017;9(2):230-238.
48. Donenberg GR, Cohen MH, Ingabire C, et al. Applying the Exploration Preparation Implementation Sustainment (EPIS) Framework to the Kigali Imbereheza Project for Rwandan Adolescents Living With HIV. *J Acquir Immune Defic Syndr.* 2019;82 Suppl 3:S289-S298.
49. Chibanda D, Mesu P, Kajawu L, Cowan F, Araya R, Abas MA. Problem-solving therapy for depression and common mental disorders in Zimbabwe: piloting a task-shifting primary mental health care intervention in a population with a high prevalence of people living with HIV. *BMC Public Health.* 2011;11:828.
50. Chibanda D, Weiss HA, Verhey R, et al. Effect of a Primary Care-Based Psychological Intervention on Symptoms of Common Mental Disorders in Zimbabwe: A Randomized Clinical Trial. *JAMA.* 2016;316(24):2618-2626.
51. Chibanda D, Verhey R, Munetsi E, Cowan FM, Lund C. Using a theory driven approach to develop and evaluate a complex mental health intervention: the friendship bench project in Zimbabwe. *Int J Ment Health Syst.* 2016;10:16.

52. The Mental Health Innovation Network. The Friendship Bench. https://www.mhinnovation.net/innovations/friendship-bench. Last accessed 17 April 2020.
53. Eaton J, McCay L, Semrau M, et al. Scale up of services for mental health in low-income and middle-income countries. *Lancet.* 2011;378(9802):1592-1603.
54. Gonzalez JS, Batchelder AW, Psaros C, Safren SA. Depression and HIV/AIDS treatment nonadherence: a review and meta-analysis. *J Acquir Immune Defic Syndr.* 2011;58(2):181-187.
55. Dow DE, Turner EL, Shayo AM, Mmbaga B, Cunningham CK, O'Donnell K. Evaluating mental health difficulties and associated outcomes among HIV-positive adolescents in Tanzania. *AIDS Care.* 2016;28(7):825-833.
56. Ramaiya MK, Sullivan KA, K OD, et al. A Qualitative Exploration of the Mental Health and Psychosocial Contexts of HIV-Positive Adolescents in Tanzania. *PLoS One.* 2016;11(11):e0165936.
57. Ramos JV, Mmbaga BT, Turner EL, et al. Modality of Primary HIV Disclosure and Association with Mental Health, Stigma, and Antiretroviral Therapy Adherence in Tanzanian Youth Living with HIV. *AIDS Patient Care STDS.* 2018;32(1):31-37.
58. Ewart CK. Social action theory for a public health psychology. *Am Psychol.* 1991;46(9):931-946.
59. Bhana A, Mellins CA, Small L, et al. Resilience in perinatal HIV+ adolescents in South Africa. *AIDS Care.* 2016;28 Suppl 2:49-59.
60. Cohen J, Mannarino A, Deblinger E. Treating Trauma and Traumatic Grief in Children and Adolescents. New York & London. The Guilford Press. 2017.
61. Young KS, Sandman CF, Craske MG. Positive and Negative Emotion Regulation in Adolescence: Links to Anxiety and Depression. *Brain Sci.* 2019;9(4).
62. Bolton P, Bass J, Neugebauer R, et al. Group interpersonal psychotherapy for depression in rural Uganda: a randomized controlled trial. *JAMA.* 2003;289(23):3117-3124.
63. Miller WR, Rollnick S. *Motivational interviewing : helping people change.* 3rd ed. New York, NY: Guilford Press; 2013.
64. Carlson RW, Crockett MJ. The lateral prefrontal cortex and moral goal pursuit. *Curr Opin Psychol.* 2018;24:77-82.
65. Ahmed SP, Bittencourt-Hewitt A, Sebastian CL. Neurocognitive bases of emotion regulation development in adolescence. *Dev Cogn Neurosci.* 2015;15:11-25.
66. Rogers, Carl R. Client-centered therapy. Boston: Houghton-Mifflin, 1951, p 64.
67. O'Donnell K, Dorsey S, Gong W, et al. Treating maladaptive grief and posttraumatic stress symptoms in orphaned children in Tanzania: group-based trauma-focused cognitive-behavioral therapy. *J Trauma Stress.* 2014;27(6):664-671.
68. AVERT. Global information and education on HIV and AIDS. Available at https://www.avert.org/professionals/hiv-around-world/sub-saharan-africa/tanzania. Last accessed 27 November 2019.
69. Nations Encyclopedia. Tanzania-Location, size, and extent. Available at: https://www.nationsencyclopedia.com/Africa/Tanzania-LOCATION-SIZE-AND-EXTENT.html. Last accessed 27 November 2019.
70. UNAIDS. United Republic of Tanzania Country Data 2018. Available at https://www.unaids.org/en/regionscountries/countries/unitedrepublicoftanzania. Last accessed 27 November 2019.
71. The PHIA Project: Tanzania Summary Sheet. Section Viral Load Suppression Among People Living With HIV, by Age and Sex, page 4. Last Accessed April 27, 2020. Accessed at https://phia.icap.columbia.edu/wp-content/uploads/2016/09/THIS_Final.pdf.

72. Population Based HIV Impact Assessment (PHIA) Project. TANZANIA HIV IMPACT SURVEY (THIS)A POPULATION-BASED HIV IMPACT ASSESSMENT 2016-2017. Available at: https://www.nbs.go.tz Last accessed 27 November 2019.
73. Rafi M. Evaluating training cascade: A methodology and case study. *Educational Research and Reviews*

. 2010;5(2):64-77.
74. Murray LK, Dorsey S, Bolton P, et al. Building capacity in mental health interventions in low resource countries: an apprenticeship model for training local providers. *Int J Ment Health Syst.* 2011;5(1):30.
75. Gearing RE, Schwalbe CS, Lee R, Hoagwood KE. The effectiveness of booster sessions in CBT treatment for child and adolescent mood and anxiety disorders. *Depress Anxiety.* 2013;30(9):800-808.
76. Ybarra ML, Korchmaros JD, Prescott TL, Birungi R. A Randomized Controlled Trial to Increase HIV Preventive Information, Motivation, and Behavioral Skills in Ugandan Adolescents. *Ann Behav Med.* 2015;49(3):473-485.
77. Moerbeek M, Teerenstra S. *Power analysis of trials with multilevel data.* Boca Raton: CRC Press, Taylor & Francis Group; 2016.
78. Gandhi M, Greenblatt RM. Hair it is: the long and short of monitoring antiretroviral treatment. *Ann Intern Med.* 2002;137(8):696-697.
79. Tabb ZJ, Mmbaga BT, Gandhi M, et al. Antiretroviral drug concentrations in hair are associated with virologic outcomes among young people living with HIV in Tanzania. *AIDS.* 2018.
80. *Dow DE, Mmbaga BT, Gallis JA, Turner EL, Gandhi M, Cunningham CK, O'Donnell K. PROMISING RESULTS FROM A PILOT RCT MENTAL HEALTH INTERVENTION FOR HIV-INFECTED YOUTH. Abstract number 2208. Conference on Retroviruses and Opportunistic Infections. March 8-11, 2020. Boston, MA, USA.*
81. Gelaye B, Williams MA, Lemma S, et al. Validity of the Patient Health Questionnaire-9 for depression screening and diagnosis in East Africa. *Psychiatry Res.* 2013;210(2):653-661.
82. Thapar A, Collishaw S, Pine DS, Thapar AK. Depression in adolescence. *Lancet.* 2012;379(9820):1056-1067.
83. Goodman R. Psychometric properties of the strengths and difficulties questionnaire. *J Am Acad Child Adolesc Psychiatry.* 2001;40(11):1337-1345.
84. Steinberg AM, Brymer MJ, Decker KB, Pynoos RS. The University of California at Los Angeles Post-traumatic Stress Disorder Reaction Index. *Curr Psychiatry Rep.* 2004;6(2):96-100.
85. Steinberg AM, Brymer MJ, Kim S, et al. Psychometric properties of the UCLA PTSD reaction index: part I. *J Trauma Stress.* 2013;26(1):1-9.
86. Spitzer RL, Kroenke K, Williams JB, Lowe B. A brief measure for assessing generalized anxiety disorder: the GAD-7. *Arch Intern Med.* 2006;166(10):1092-1097.
87. Carver, C. S. (1997). You want to measure coping but your protocol's too long: Consider the Brief COPE. International Journal of Behavioral Medicine, 4, 92-100.
88. Gottert A, Friedland B, Geibel S, et al. The People Living with HIV (PLHIV) Resilience Scale: Development and Validation in Three Countries in the Context of the PLHIV Stigma Index. *AIDS Behav.* 2019;23(Suppl 2):172-182.
89. Wright K, Naar-King S, Lam P, Templin T, Frey M. Stigma scale revised: reliability and validity of a brief measure of stigma for HIV+ youth. *J Adolesc Health.* 2007;40(1):96-98.
90. Tesfaye M, Olsen MF, Medhin G, Friis H, Hanlon C, Holm L. Adaptation and validation of the short version WHOQOL-HIV in Ethiopia. *Int J Ment Health Syst.* 2016;10:29.

91. WHO Multi-Country Study on Women's Health and Domestic Violence: Core Questionnaire and WHO Instrument. 2003, Version 10. Geneva, Switzerland: World Health Organisation.
92. Carey MP, Schroder KE. Development and psychometric evaluation of the brief HIV Knowledge Questionnaire. *AIDS Educ Prev.* 2002;14(2):172-182.
93. Wilson IB, Lee Y, Michaud J, Fowler FJ, Rogers WHJA, Behavior. Validation of a new three-item self-report measure for medication adherence. 2016;20(11):2700-2708.
94. Tabb ZJ, Mmbaga BT, Gandhi M, et al. Antiretroviral drug concentrations in hair are associated with virologic outcomes among young people living with HIV in Tanzania. *AIDS.* 2018;32(9):1115-1123.
95. Kauermann G, Carroll RJ. A note on the efficiency of sandwich covariance matrix estimation. *J Am Stat Assoc.* 2001;96(456):1387-1396.
96. Roberts C, Roberts SA. Design and analysis of clinical trials with clustering effects due to treatment. *Clin Trials.* 2005;2(2):152-162.
97. Lu K. On efficiency of constrained longitudinal data analysis versus longitudinal analysis of covariance. *Biometrics.* 2010;66(3):891-896.
98. Li P, Redden DT. Comparing denominator degrees of freedom approximations for the generalized linear mixed model in analyzing binary outcome in small sample cluster-randomized trials. *BMC Med Res Methodol.* 2015;15(1):38.
99. Preacher KJ, Hayes AF. Asymptotic and resampling strategies for assessing and comparing indirect effects in multiple mediator models. *Behav Res Methods.* 2008;40(3):879-891.
100. Ulin PR, Robinson ET, Tolley EE. (2005). Qualitative Methods in Public Health: A field guide for applied research. San Francisco CA: Jossey-Bass.; .
101. Farmer T, Robinson K, Elliott SJ, Eyles J. Developing and implementing a triangulation protocol for qualitative health research. *Qual Health Res.* 2006;16(3):377-394.
102. O'Cathain A, Murphy E, Nicholl J. Three techniques for integrating data in mixed methods studies. *BMJ.* 2010;341:c4587.

**SYV: A Mental Health Intervention to Improve HIV Outcomes in Tanzanian Youth**,
Version 2: July 28, 2022

## Appendix I: Quantitative Survey
